# Supplementary material for: Multi-omic spatial effects on high-resolution AI-derived retinal thickness
Source: Nat Commun. 2025 Feb 4;16:1317. doi: 10.1038/s41467-024-55635-7 (PMC11794613; doi:10.1038/s41467-024-55635-7)
Supplement: Supplementary file 1 — Supplementary Information [file 41467_2024_55635_MOESM1_ESM.pdf]

Supplementary Information

Supplementary Figure Index

Supplementary Figure 1. Sample A* segmentations showing potential problems with directly applying A* to the UKBiobank dataset
Supplementary Figure 2. Quality exclusion criterion 1 - faint B-scans
Supplementary Figure 3. Quality exclusion criterion 2 - Thickness measurements too thin in scan region
Supplementary Figure 4. Quality exclusion criterion 3 - Thickness too thin at specific locations
Supplementary Figure 5. Quality exclusion criterion 4 - Discontinuities in ILM-RPE segmentation
Supplementary Figure 6. Quality exclusion criterion 5 - Location thickness discontinuities
Supplementary Figure 7. Quality exclusion criterion 6 - undocumented retinal disease
Supplementary Figure 8. Early treatment of diabetic retinopathy (ETDRS) grid with orientation used for all retinal images
Supplementary Figure 9. Secondary quality control filtering of the aligned OCT data
Supplementary Figure 10. Two dimensional functional principal component analysis
Supplementary Figure 11: Retinal Thickness FPC representations
Supplementary Figure 12. Summary of RT measures.
Supplementary Figure 13. Pixel-level associations with basic characteristics
Supplementary Figure 14. LD-score regression intercepts
Supplementary Figure 15. Procedure for collation of GWAS results
Supplementary Figure 16. Summary of genetic loci
Supplementary Figure 17. Heritability estimates
Supplementary Figure 18. Summary of loci identified in present analyses, compared to previous GWAS of RT phenotypes.
Supplementary Figure 19. Comparison of effect sizes and p-values in current versus previous GWAS of RT.
Supplementary Figure 20. Clustering of SNPs identified through the pixel-level analyses, based on effects across all 29,041 pixels
Supplementary Figure 21. Clustering of pixels, based on SNP effects for loci identified through the pixel-level analyses
Supplementary Figure 22. Cross ancestry comparisons
Supplementary Figure 23. Top 30 traits by number of SNP associations from PheWAS
Supplementary Figure 24. Genetic correlations
Supplementary Figure 25. Gene Ontology Over-Representation Analysis
Supplementary Figure 26. Smoking sensitivity analyses
Supplementary Figure 27. Pixel-level metabolite associations
Supplementary Figure 28. BMI sensitivity analyses for metabolite associations
Supplementary Figure 29. Metabolite associations with FPCs
Supplementary Figure 30. Over-representation analysis on age-interaction metabolite results
Supplementary Figure 31. Metabolite Genetic Score associations
Supplementary Figure 32. Smoking sensitivity analysesfor PheCode associations
Supplementary Figure 33. Disease and quantitative trait Genetic Score associations
Supplementary Figure 34. Associations with blood cell traits and inflammation markers
Supplementary Figure 35. Distributions of 100 randomly selected pixels

Supplementary Figure 36: Pearson's moment coefficient of skewness for each pixel across the scan
Supplementary Figure 37: Sensitivity analyses for pixel-level RT trait transformations

Supplementary Table Index

Supplementary Table 1. Available OCT data, and participant exclusions
Supplementary Table 2. Basic characteristics for the analysed cohort
Supplementary Table 3. Associations between RT fPCs and basic characteristics
Supplementary Table 4. Sample sizes for -omic association analyses

Supplementary Figures

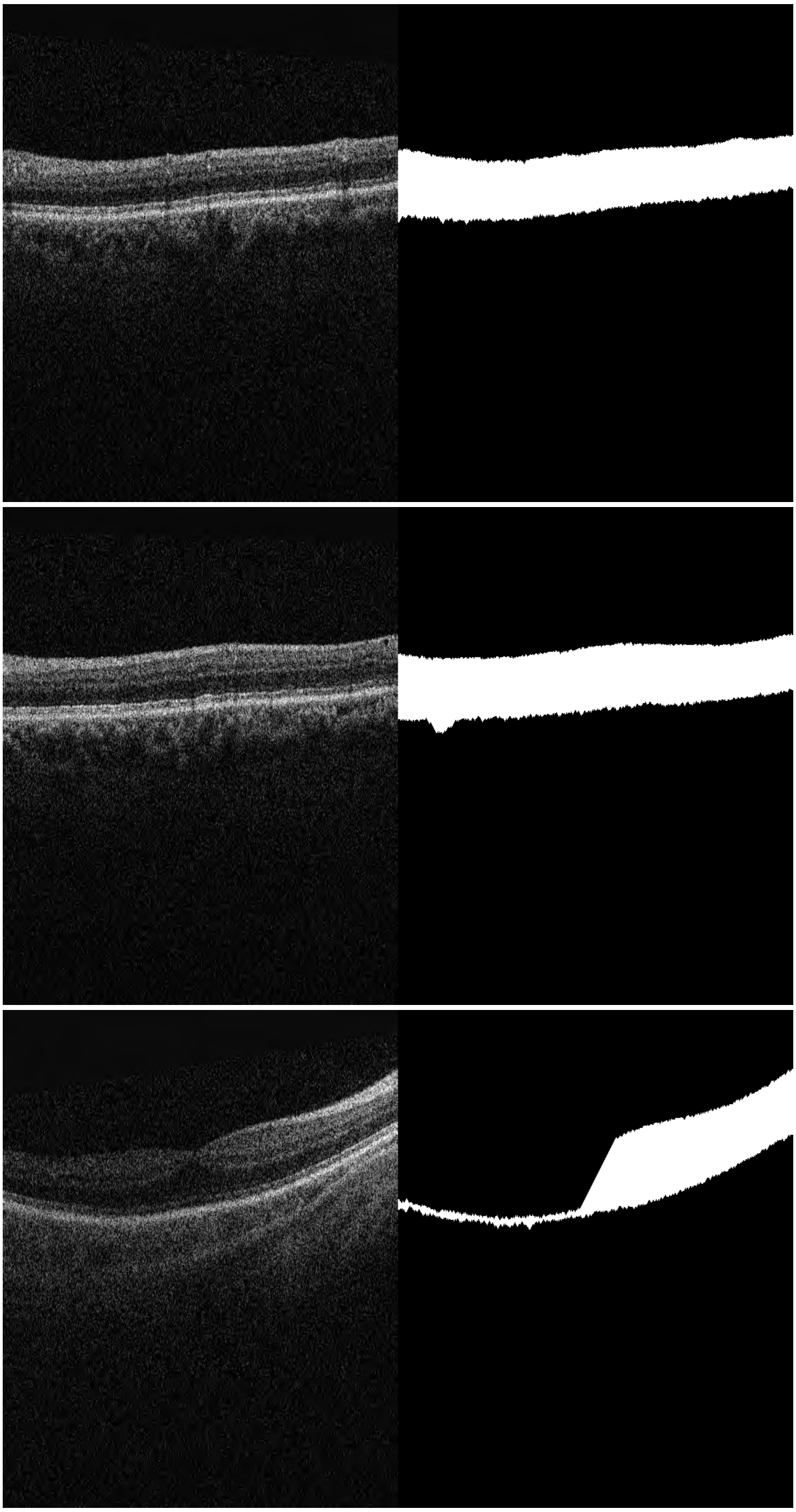

Supplementary Figure 1: Example A* segmentations with the raw B-scan on the left and the A* segmentations on the right. A* does well in some cases such as the top pair (**a**), but can fail locally in the middle pair or catastrophically in the last pair (**b** and **c**). Scans reproduced by kind permission of UK Biobank ©.

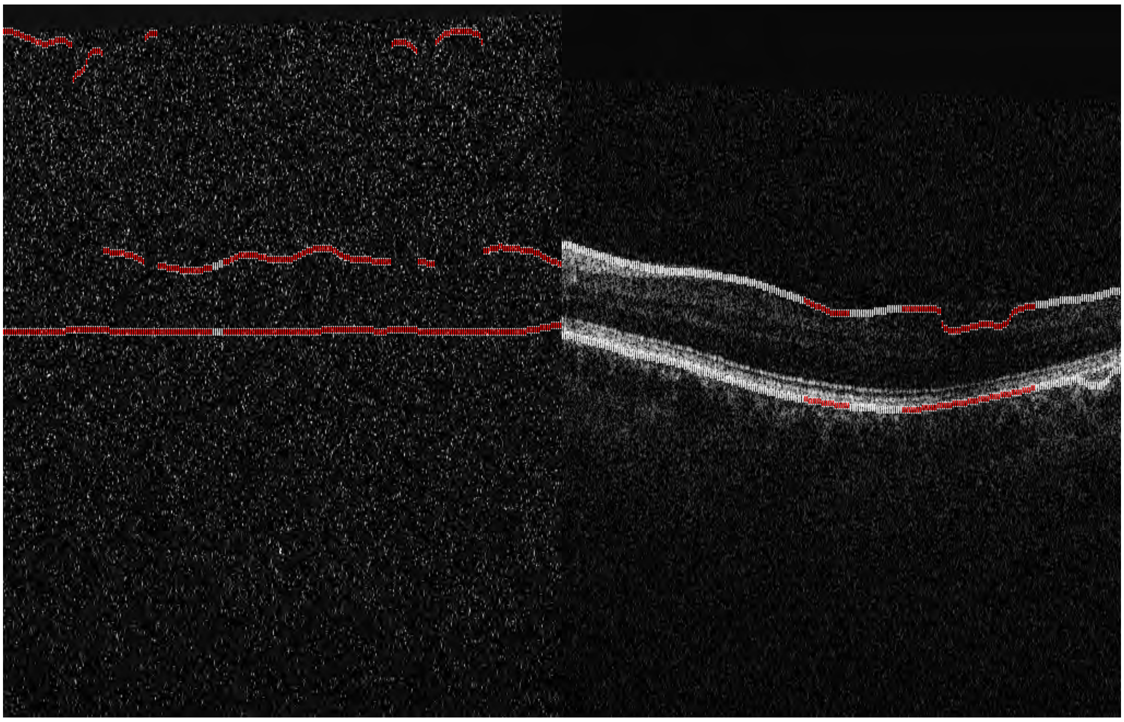

Supplementary Figure 2. Quality exclusion criterion 1 - faint B-scans. **a.** Too faint often happens when no retina is imaged, **b.** Some parts of the retina are too faint. Scans reproduced by kind permission of UK Biobank ©.

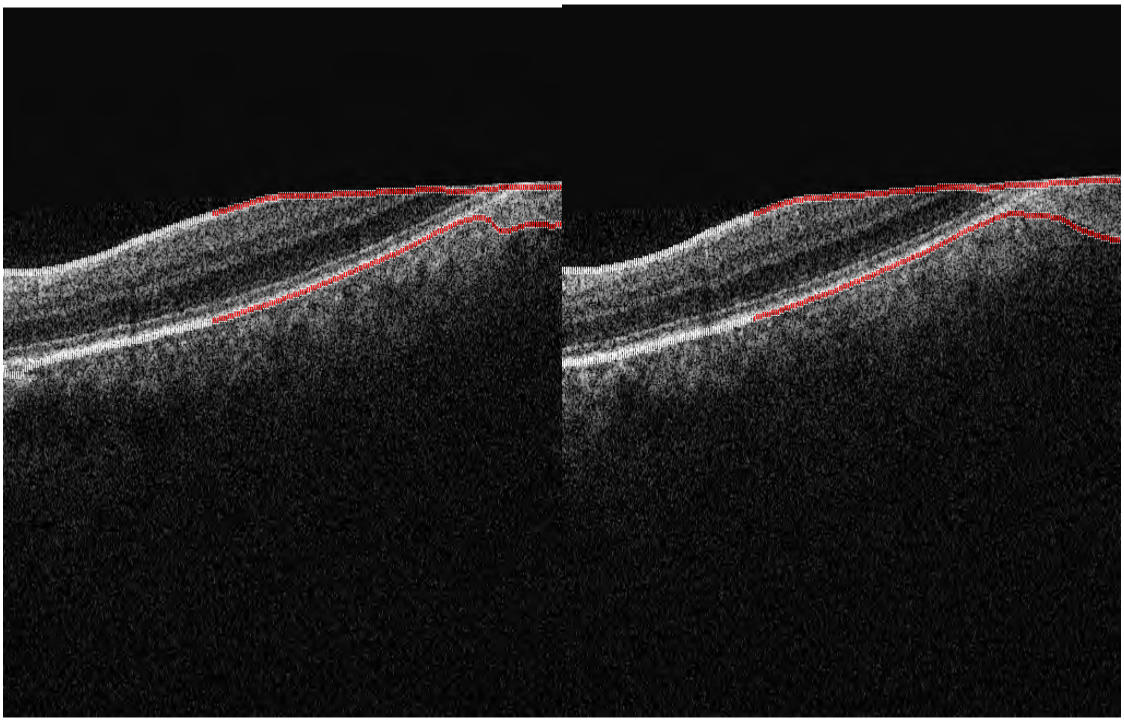

Supplementary Figure 3. Quality exclusion criterion 2 - Thickness measurements too thin in scan region. **a.** and **b.** The retina is too thin when it is cut off. Scans reproduced by kind permission of UK Biobank ©.

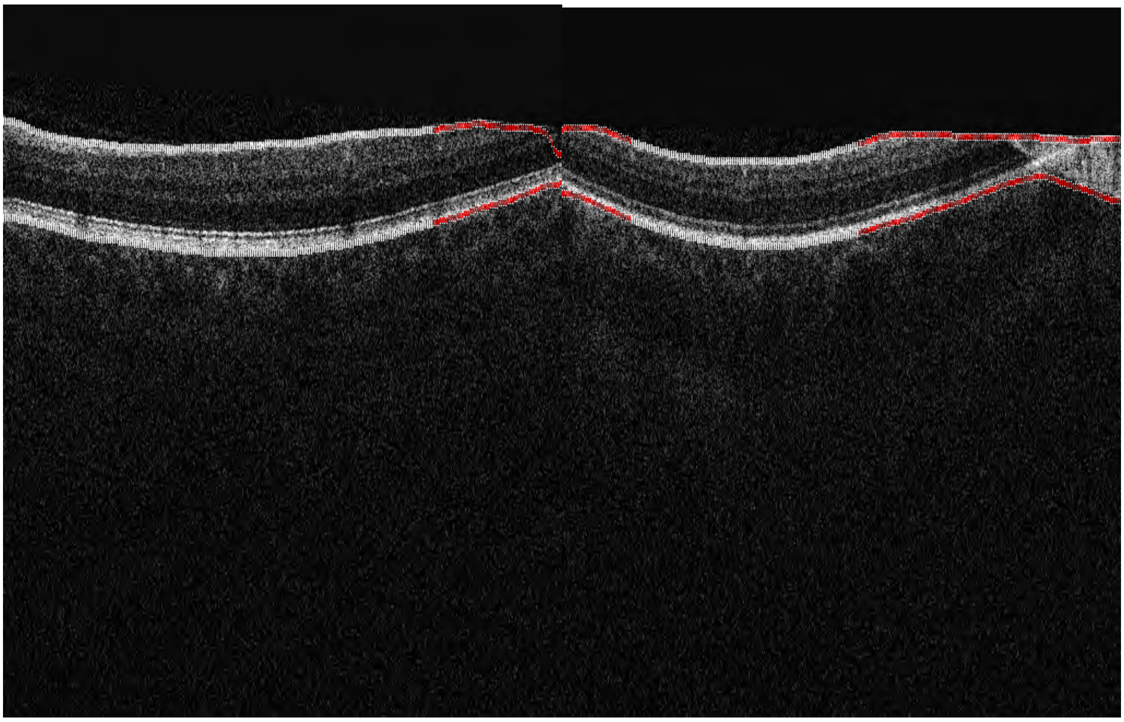

Supplementary Figure 4. Quality exclusion criterion 3 - thickness too thin at specific locations. **a.** and **b.** When the retina is cut off, there is no background reflectivity near the ILM or RPE layer boundaries. Scans reproduced by kind permission of UK Biobank ©.

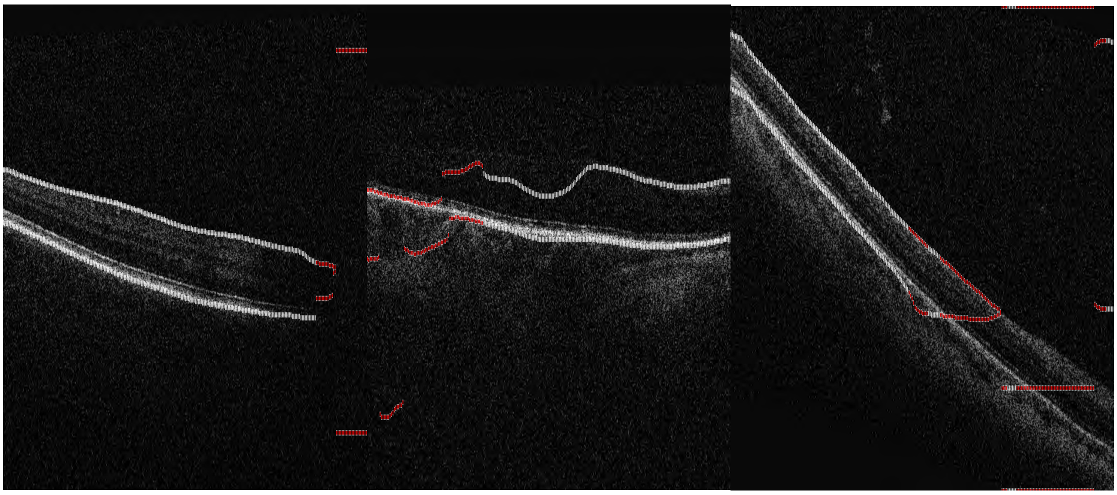

Supplementary Figure 5. Quality exclusion criterion 4 - Discontinuities in ILM-RPE segmentation. **Figures a** and **b** are due to OCT quality and **c** due to algorithm failures. Scans reproduced by kind permission of UK Biobank ©.

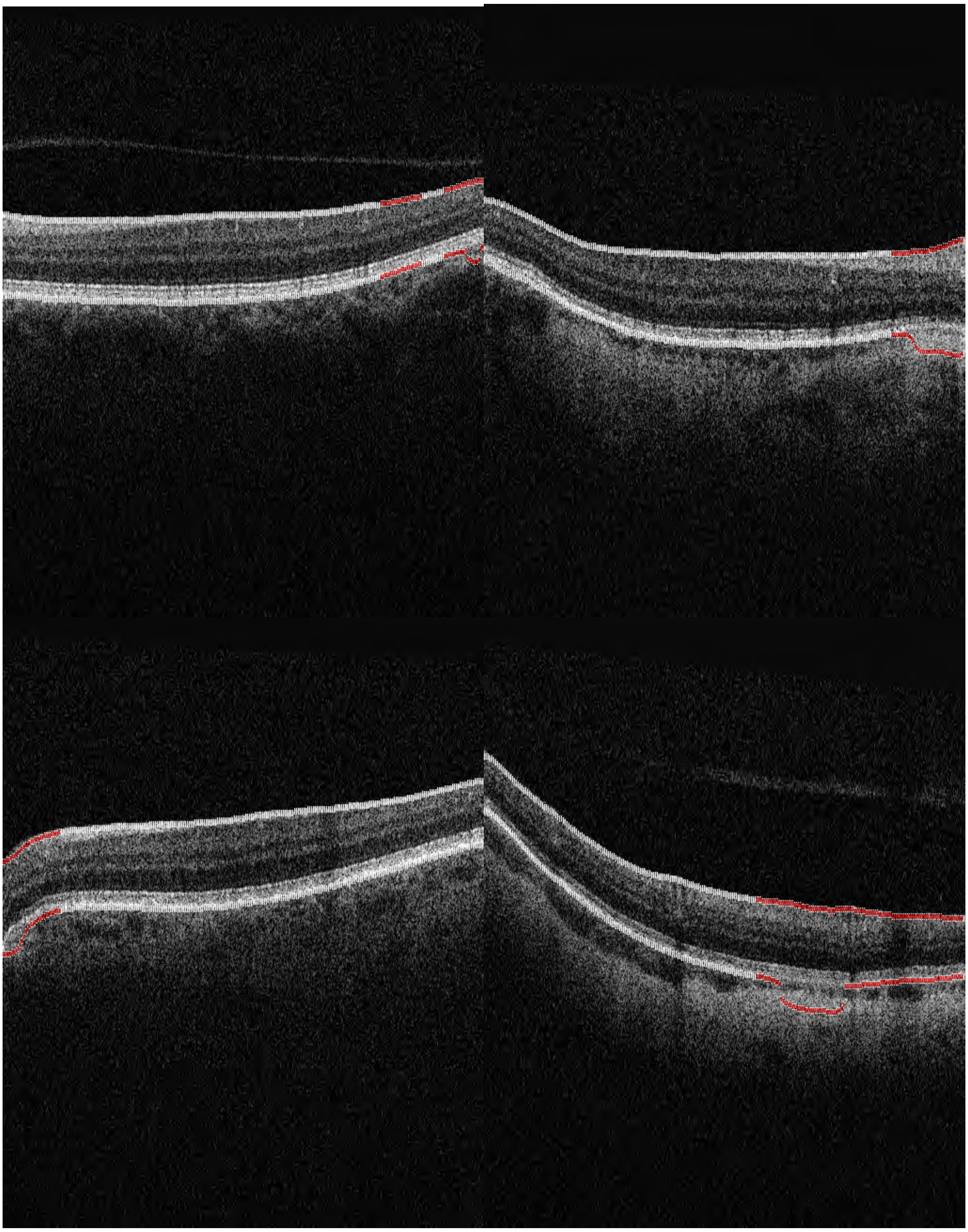

Supplementary Figure 6. Quality exclusion criterion 5 - Location thickness discontinuities. **a-d** show examples of anomalies detected by local variability in the ILM-RPE segmentation. Scans reproduced by kind permission of UK Biobank ©.

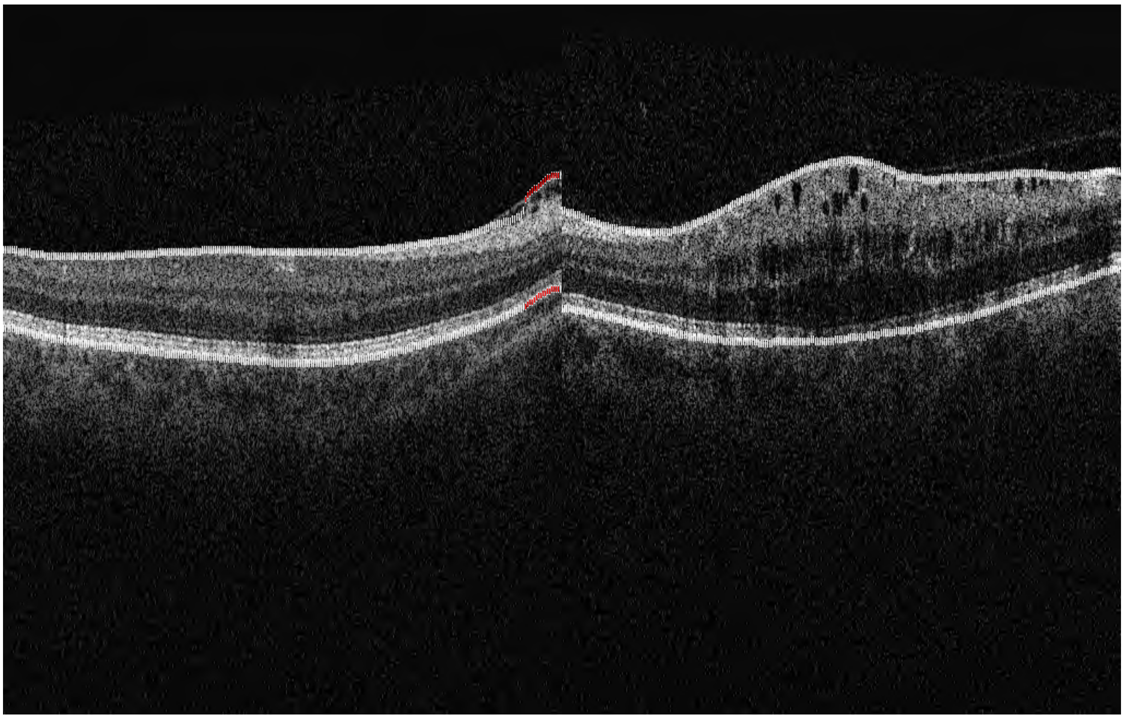

Supplementary Figure 7. Quality exclusion criterion 6 - undocumented retinal disease. **a** and **b** show examples of diseased retinas flagged by the quality control step. Example **a** was flagged by large local deviations, and **b** by too thick a retina due to gaps in the retinal layers. Scans reproduced by kind permission of UK Biobank ©.

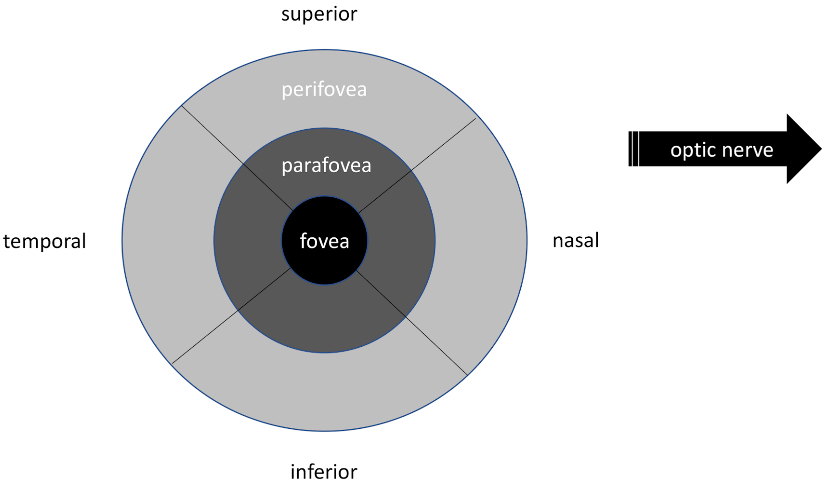

Supplementary Figure 8. Early treatment of diabetic retinopathy (ETDRS) grid with orientation used for all retinal images.

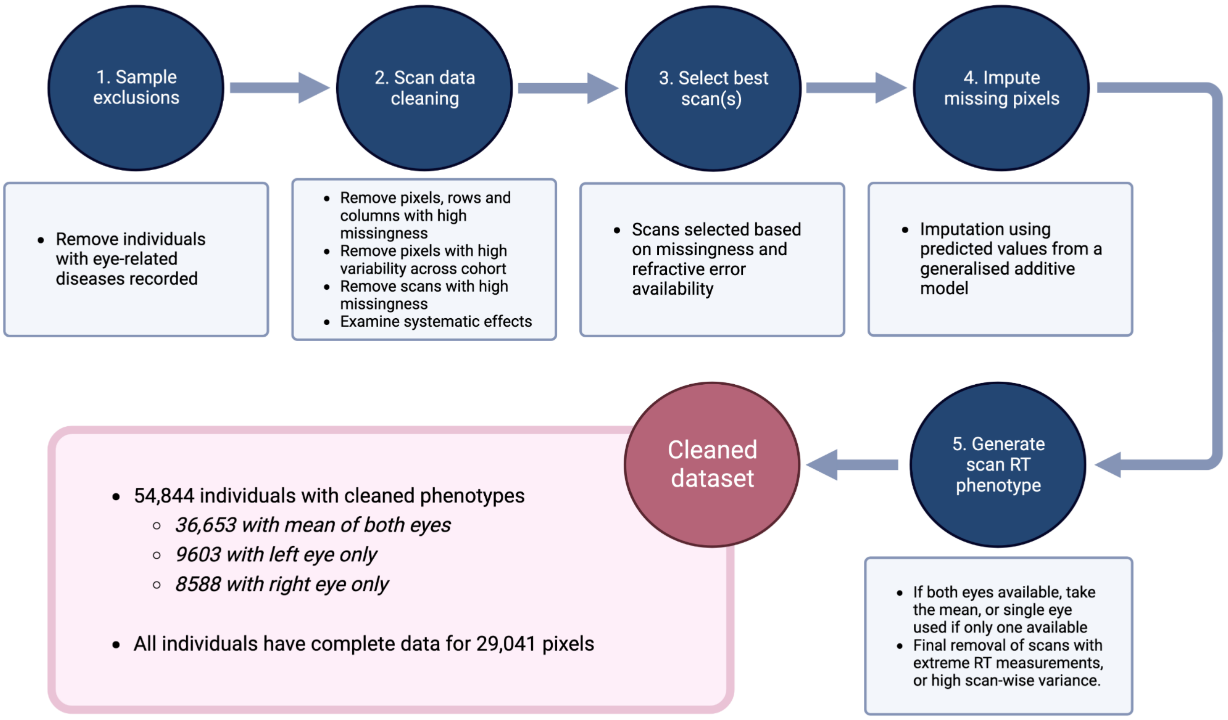

Supplementary Figure 9. Secondary quality control filtering of the aligned OCT data. Depiction of quality control filtering at both individual and pixel levels. Created in https://BioRender.com. Jackson, V. (2024) https://BioRender.com/x96p715

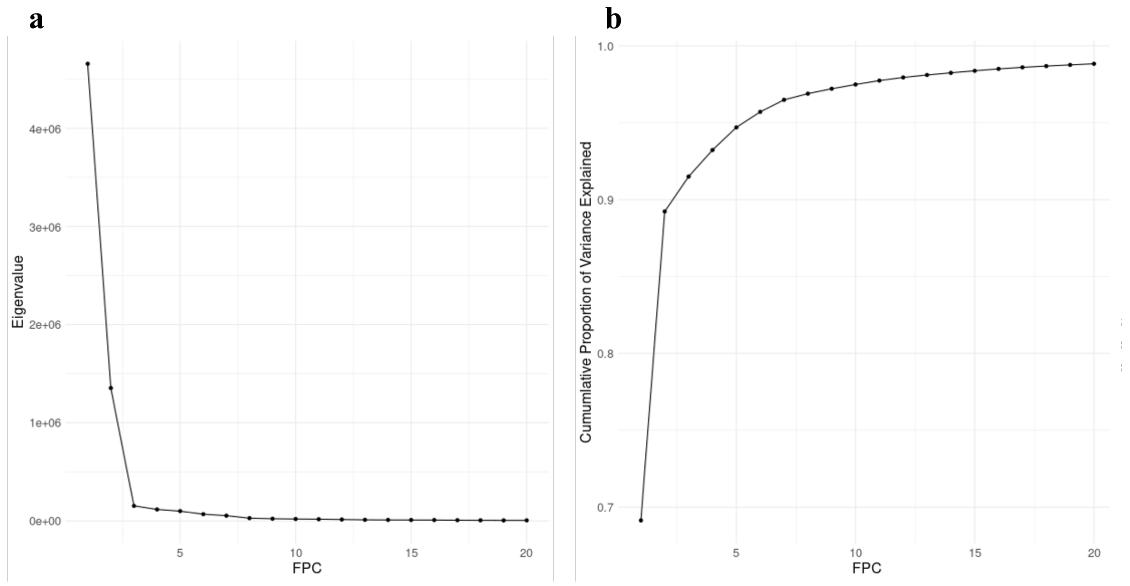

Supplementary Figure 10. Scree plot and variation explained by functional principal component analysis (FPCA). Scree plot (**a**), and plot of cumulative variance explained (**b**) for RT data for FPC dimensionality reduction approach. Six FPCs capture 95% of all of the RT variation.

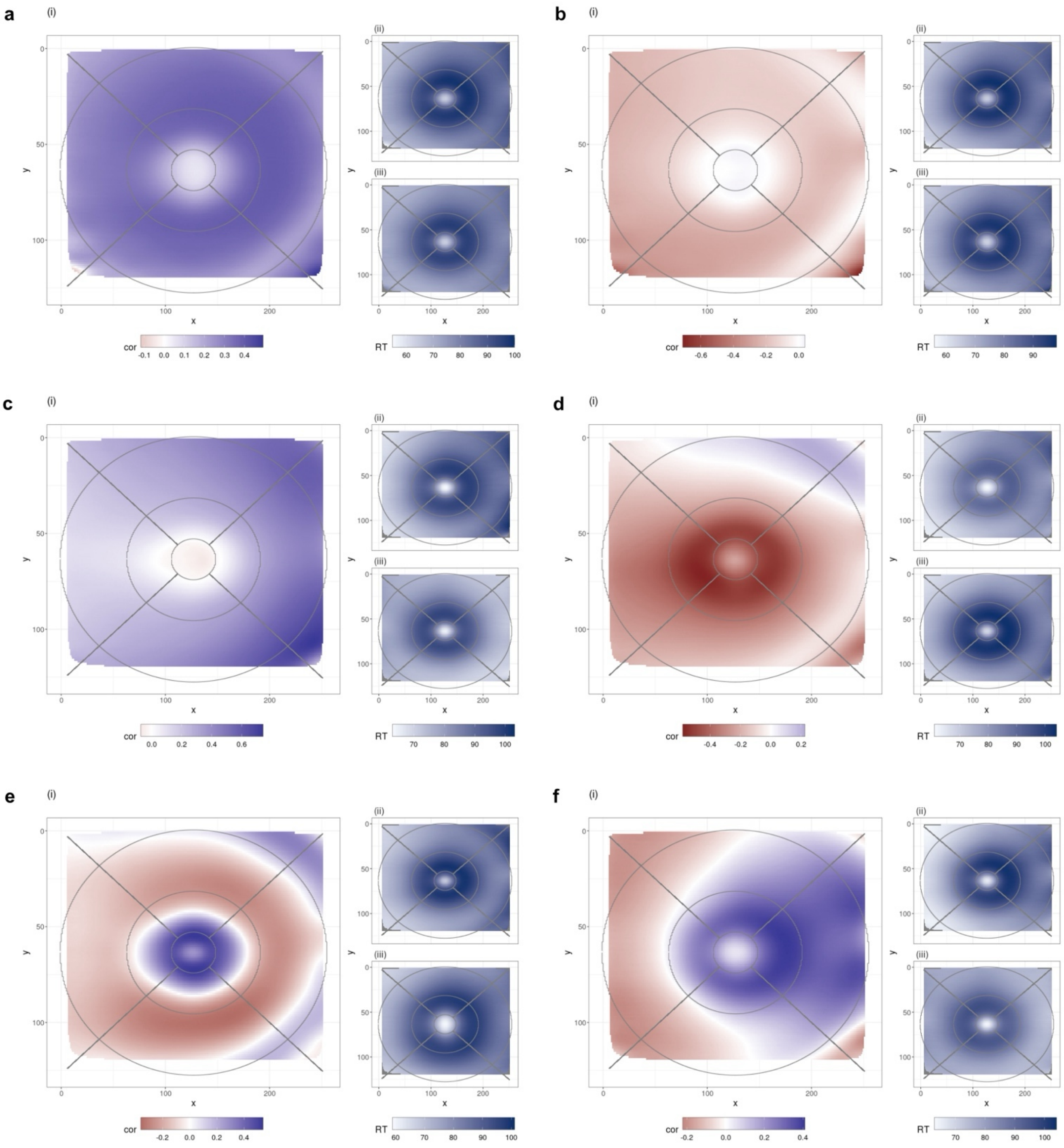

Supplementary Figure 11: Retinal Thickness FPC representations.
For each FPC 1-6 (A-F): (i) shows the Pearson correlation coefficent (cor) between individual FPC scores and each pixel wise RT value. Higher absolute correlations indicate a greater contribution to the FPC for that pixel. (ii) and (iii) show the averaged RT scan for the 100 individuals with the (ii) highest and (iii) lowest score values for that FPC, giving an idea of the dynamic range for the RT spatial patterning captured by each FPC.

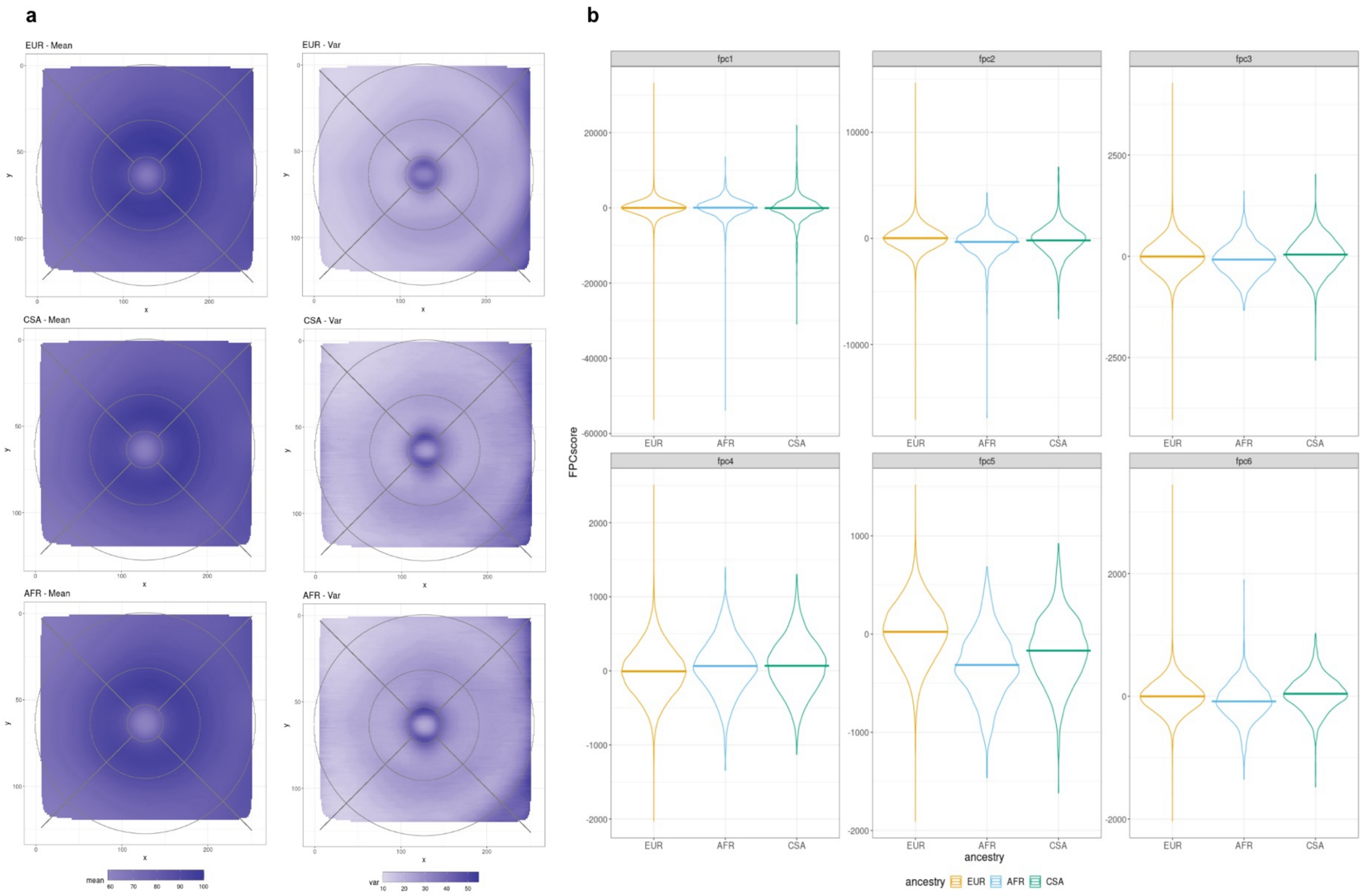

Supplementary Figure 12. Summary of RT measures.
Summary of A. pixel-level, and B. FPCs of RT measures, stratified by ancestry. A. left plot shows mean; right plot show variance, per pixel. B. Violin plots for each FPC showing the density distributions for each ancestry. Horizontal line corresponds with median FPC value.
EUR – European (n=43,148); AFR – African (n=1,179); CSA – Central and South Asian (n=1,161); Var - variance.

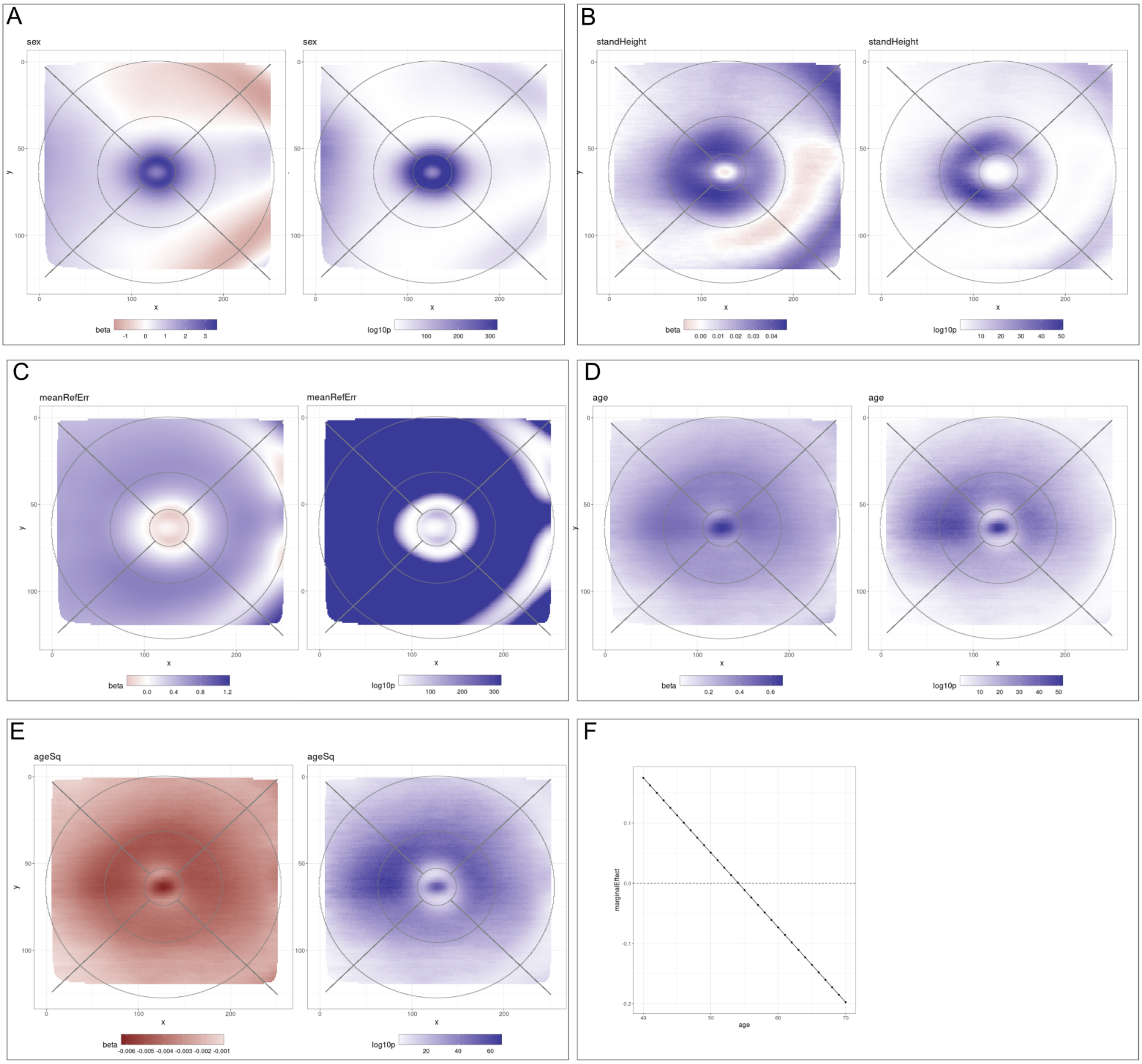

Supplementary Figure 13: Pixel-level associations with basic characteristics (effect, left; -log10 p-value, right). P-values based on a two-sided t-test, for the corresponding beta in the linear regression. Pixel-level associations with **A**) sex **B**) standing Height **C**) refractive error (spherical equivalent), **D**) age and **E**) age-squared. **F**) shows the marginal effect (point estimate) of age for central foveal pixel (64_128).

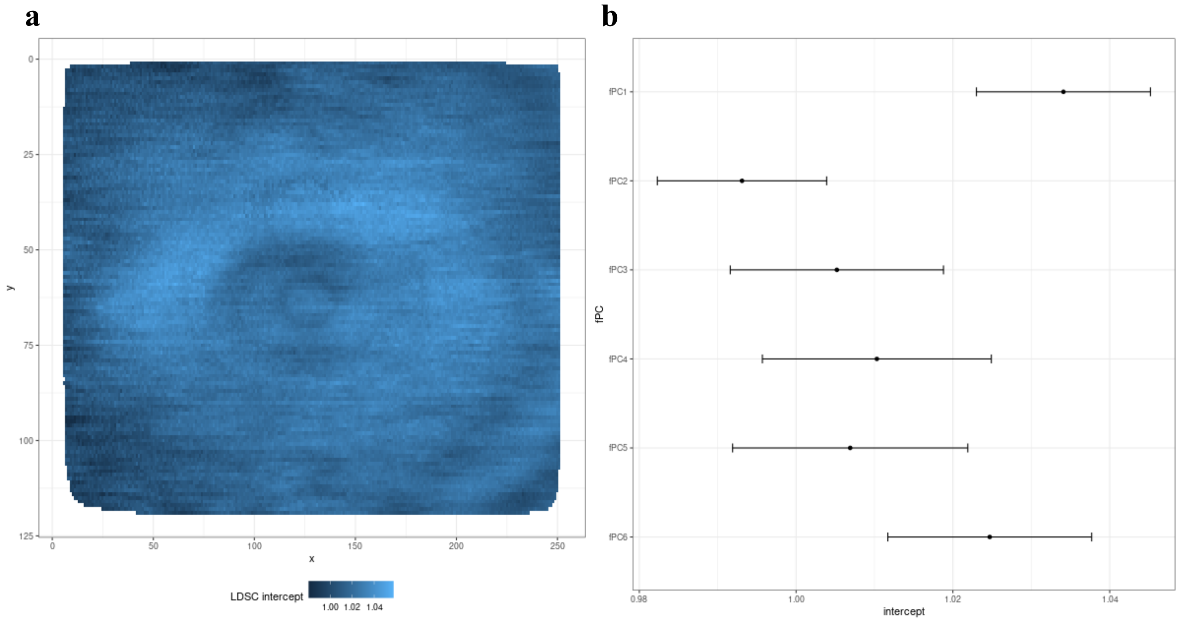

Supplementary Figure 14: a) LD-score intercepts for each pixel, **b**) LD-score intercepts (points), with standard errors (bars), for FPCs 1 to 6.

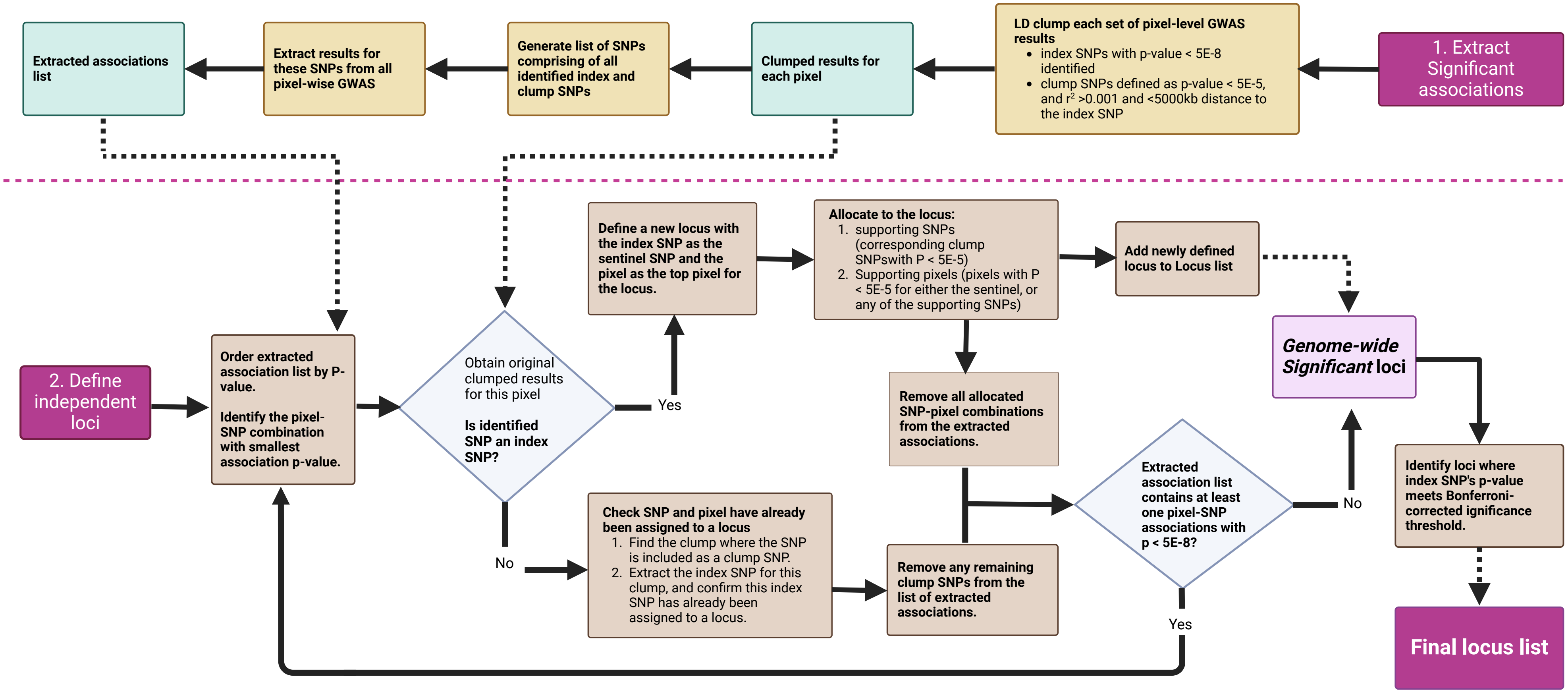

Supplementary Figure 15. Procedure for collation of GWAS results. Flowchart of iterative procedure developed to summarise GWAS results from all 29,041 RT pixels. Created in BioRender. Jackson, V. (2024) https://BioRender.com/p01e985

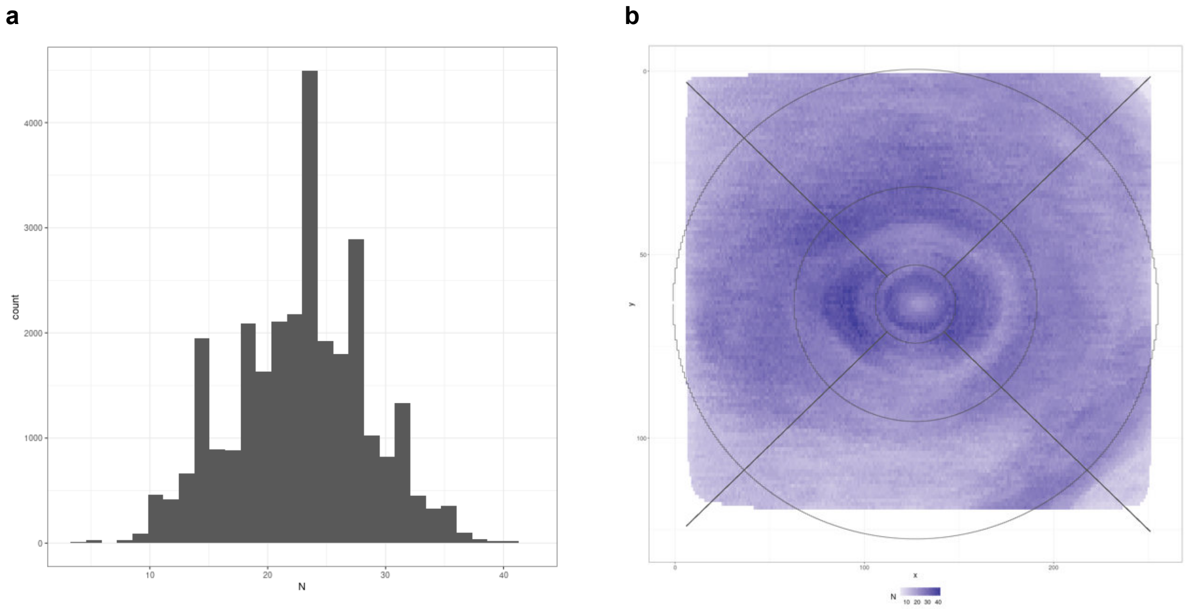

Supplementary Figure 16. Summary of genetic loci
(a) Histogram and (b) spatial distribution, of the count of Bonferroni-corrected significant loci (p<5e-08/29,041) for the pixel-level GWAS results.

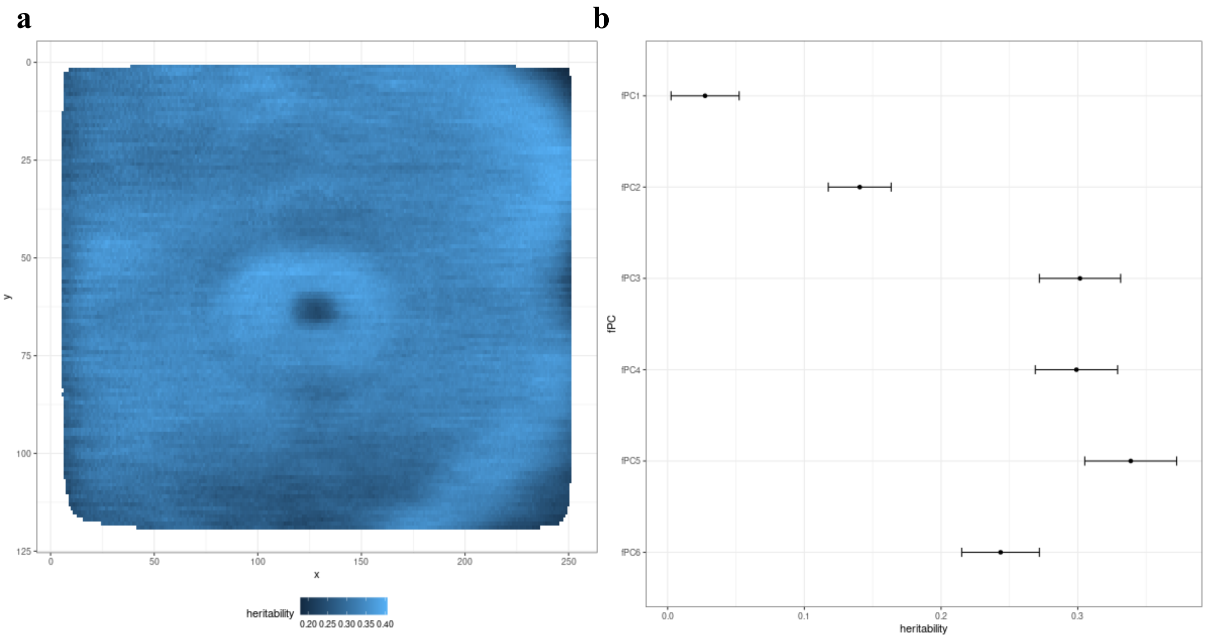

Supplementary Figure 17: a) Heritability estimates for each pixel, **b**) Heritability estimates (points), with standard errors (bars), for FPCs 1 to 6.

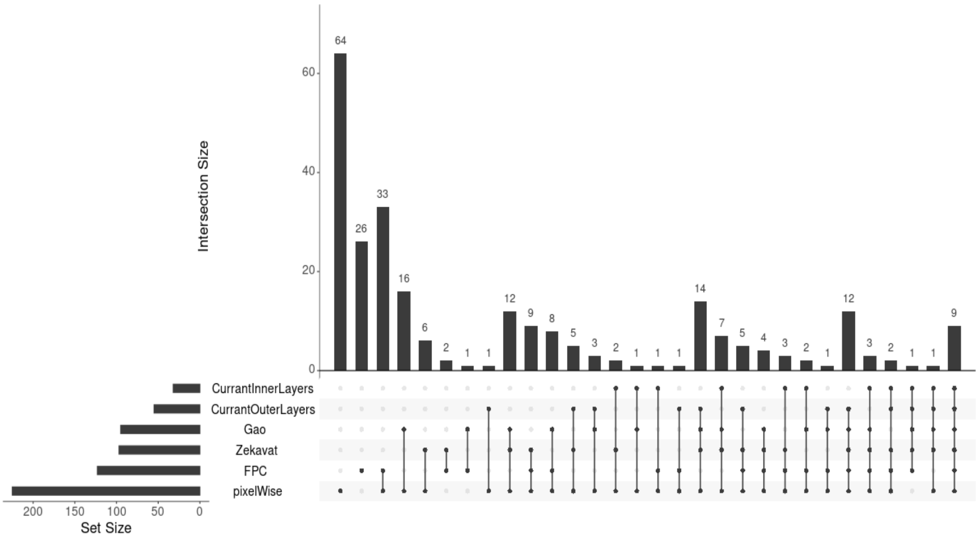

Supplementary Figure 18. Summary of loci identified in present analyses, compared to previous GWAS of RT phenotypes.

Comparison of loci identified through the pixel-level and/or FPC analyses, with loci identified in previous GWAS of RT measures (CurrantInnerLayers[1]; CurrantOuterLayers[2]; Gao[3]; Zekavat[4]), displayed as an upsetR plot. For these annotations, the sentinel SNP along with all other SNPs allocated to each locus were compared to previously reported SNPs. Plot only includes loci meeting P<5e-8 in the present analyses.

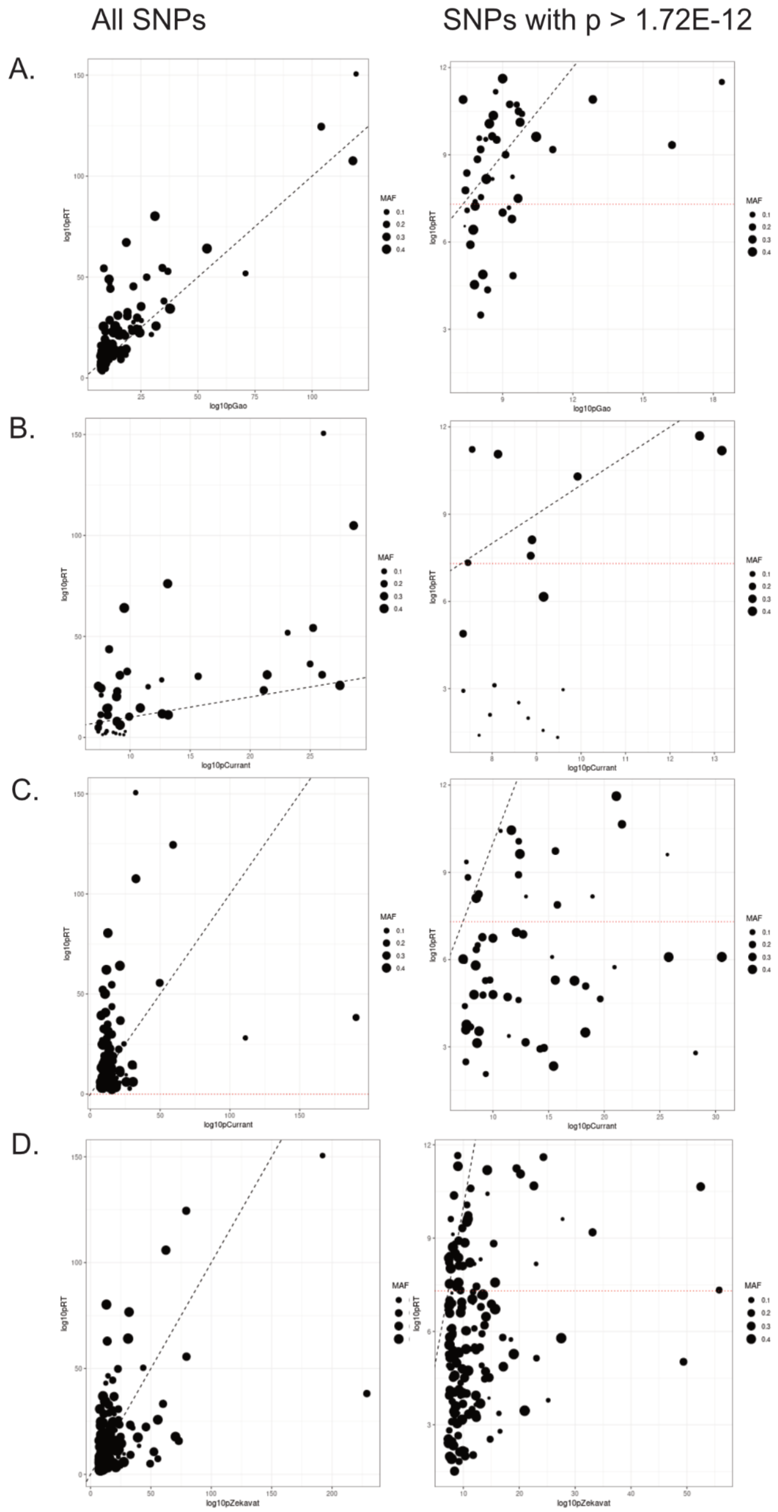

Supplementary Figure 19. Comparison of effect sizes and p-values in current versus previous GWAS of RT.

Shown are comparison of effect sizes for SNPs identified in previous studies (**A**[3]; **B**.[1]**C**.[2] **D**.[4], vs the smallest p-value from the pixel-level or fPC GWAS. Data points are sized based on SNP MAF. The left pot shows all SNPs. The right plot shows SNPs not meeting the pixel-level Bonferroni adjusted significance level. The red dotted line shows the standard genome-wide significance threshold (P<5E-8). P-values based on a two-sided t-test, for the SNP beta in the linear regression.

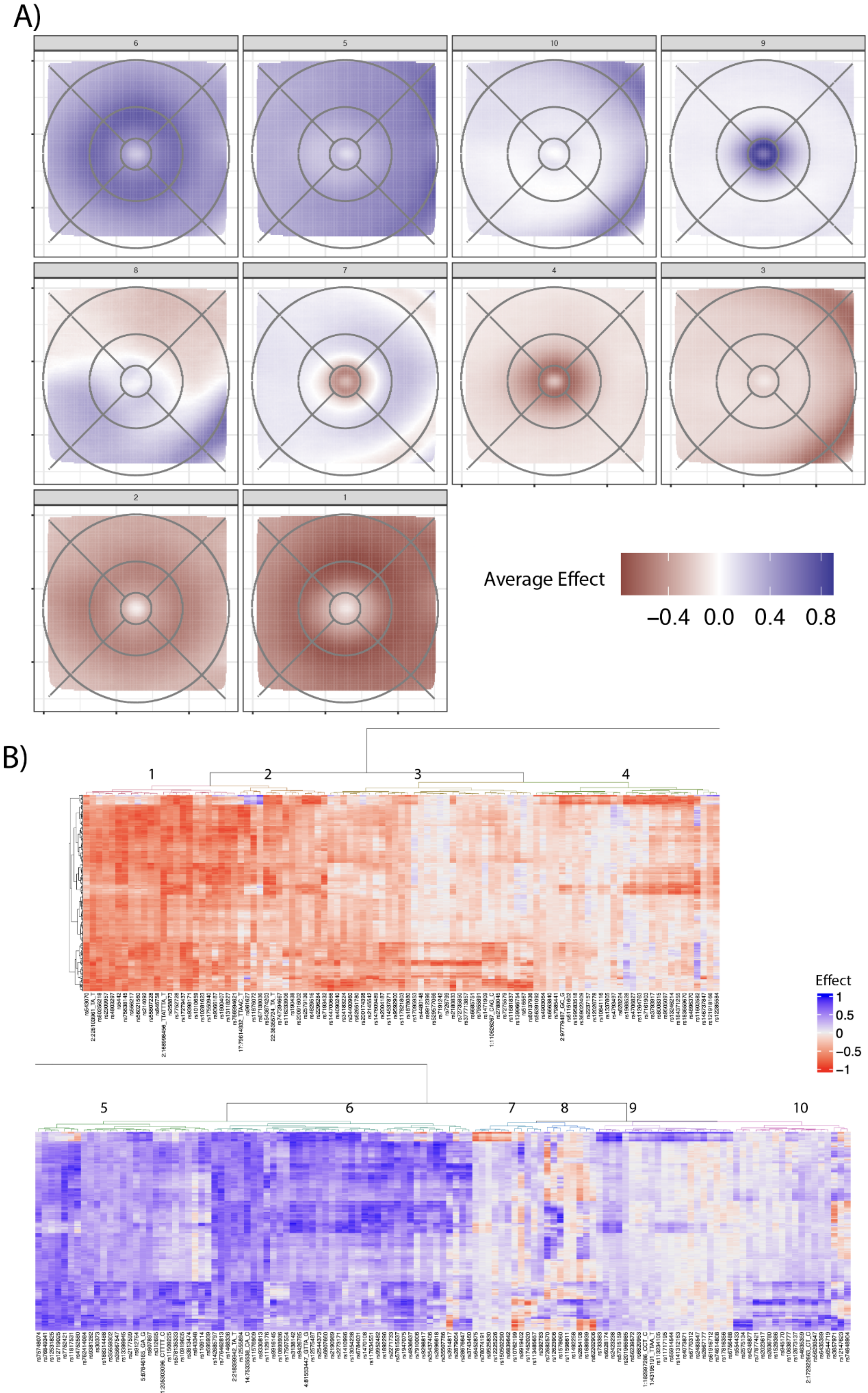
A)
6
5
10
9
8
7
4
3
2
1
Average Effect
−0.4 0.0 0.4 0.8
B)
1
2
3
4
5
6
7
8
9
10
Effect
1
0.5
0
−0.5
−1

Supplementary Figure 20. Unsupervised hierarchical clustering of SNPs identified through the pixel-level analyses, based on effects across all 29,041 pixels. **A)** Pixel-wise average effect across SNPs within each detected cluster. **B**) Heatmap showing SNPs clusters and their effect estimates (betas) on a random subset of pixels. SNPs are annotated by cluster in Supplementary Data 1.

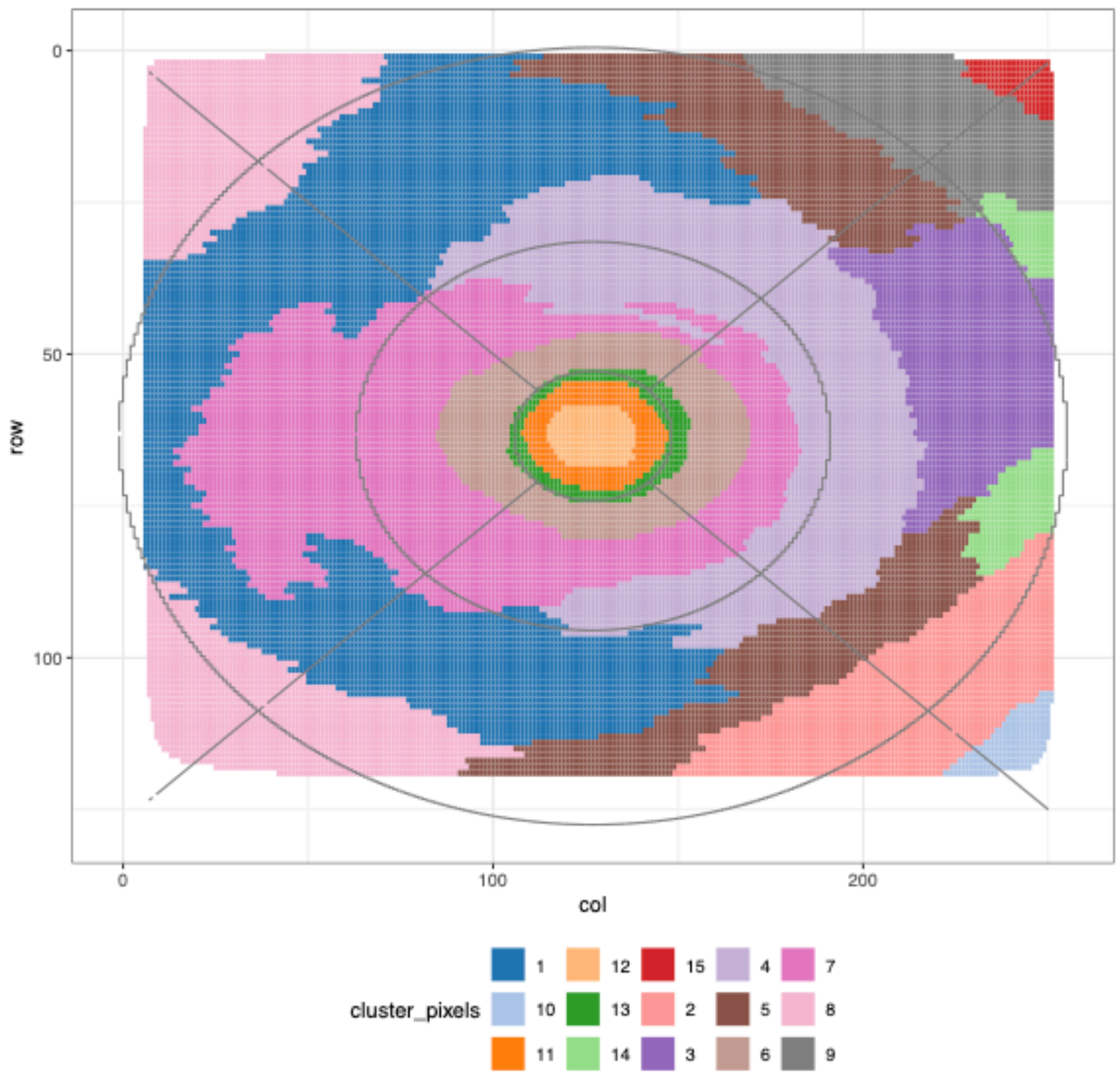

Supplementary Figure 21. Clustering of pixels, based on SNP effects for loci identified through the pixel-level analyses. In this figure, every pixel is colored according to the cluster it was assigned to in the unsupervised hierarchical clustering analysis. The ETDRs grid is also presented to ease interpretation of the clusters' locations.

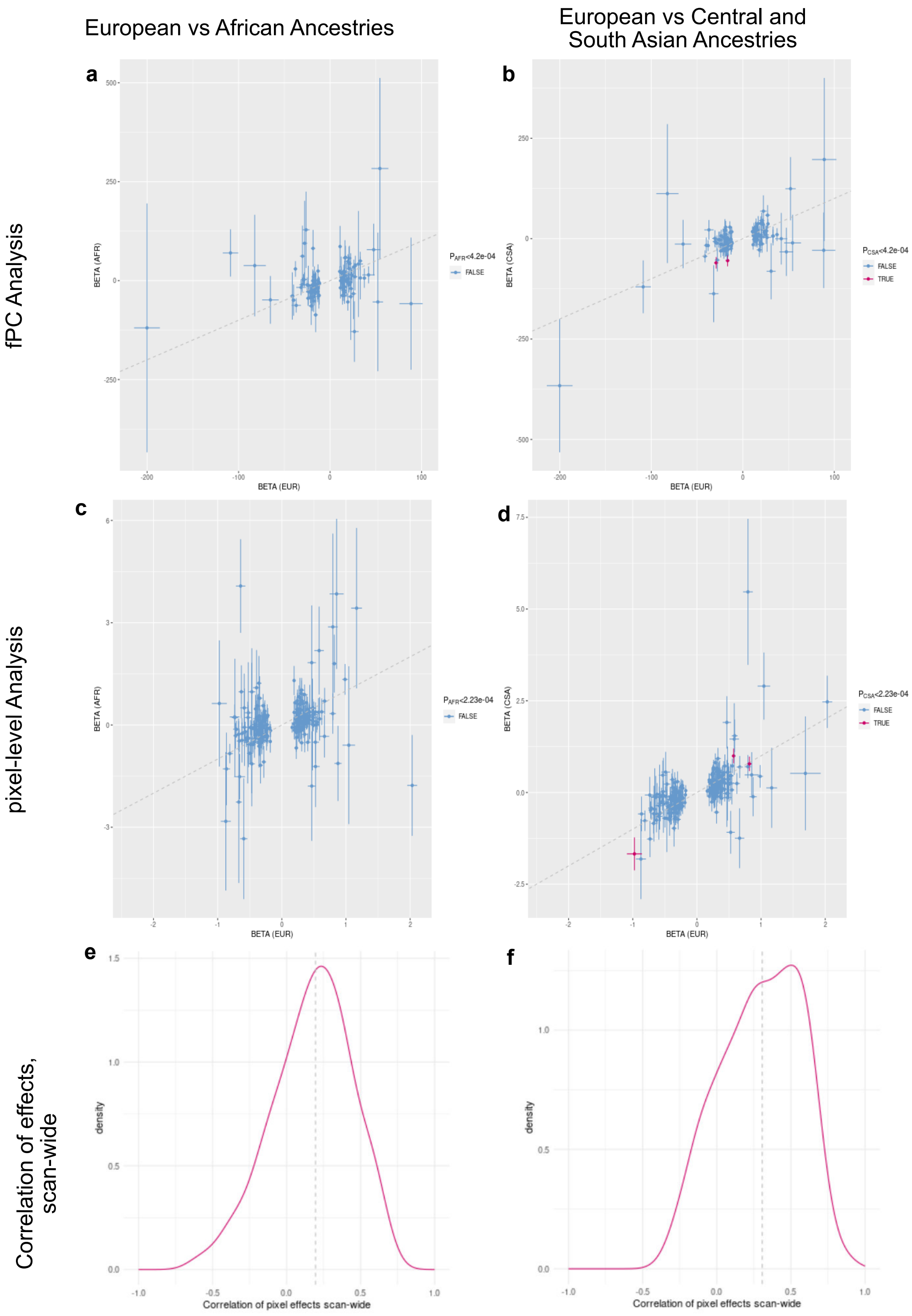

Supplementary Figure 22. Cross ancestry comparisons
Cross ancestry genetic effect size comparisons of effect estimates for EUR vs AFR (A, C, E) and EUR vs CSA (B, D, F). A-D show comparisons of effect sizes (points), with standard errors (bars), from genetic association analyses for the sentinel SNP and the top pixel/FPC, for all loci identified through FPC (A and B) and pixel-level (C and D) analyses, detected in the EUR discovery analyses. Loci meeting the Bonferroni corrected significance threshold in the AFR/CSA individuals for each analysis indicated in red. E and F show the distribution of the scan-wide correlations of effect sizes (ie the correlation of effects across all pixels), for each sentinel SNP identified through the pixel-level analysis. The dotted line shows the median scan-wide correlation. EUR – European; AFR – African; CSA – Central and South Asian

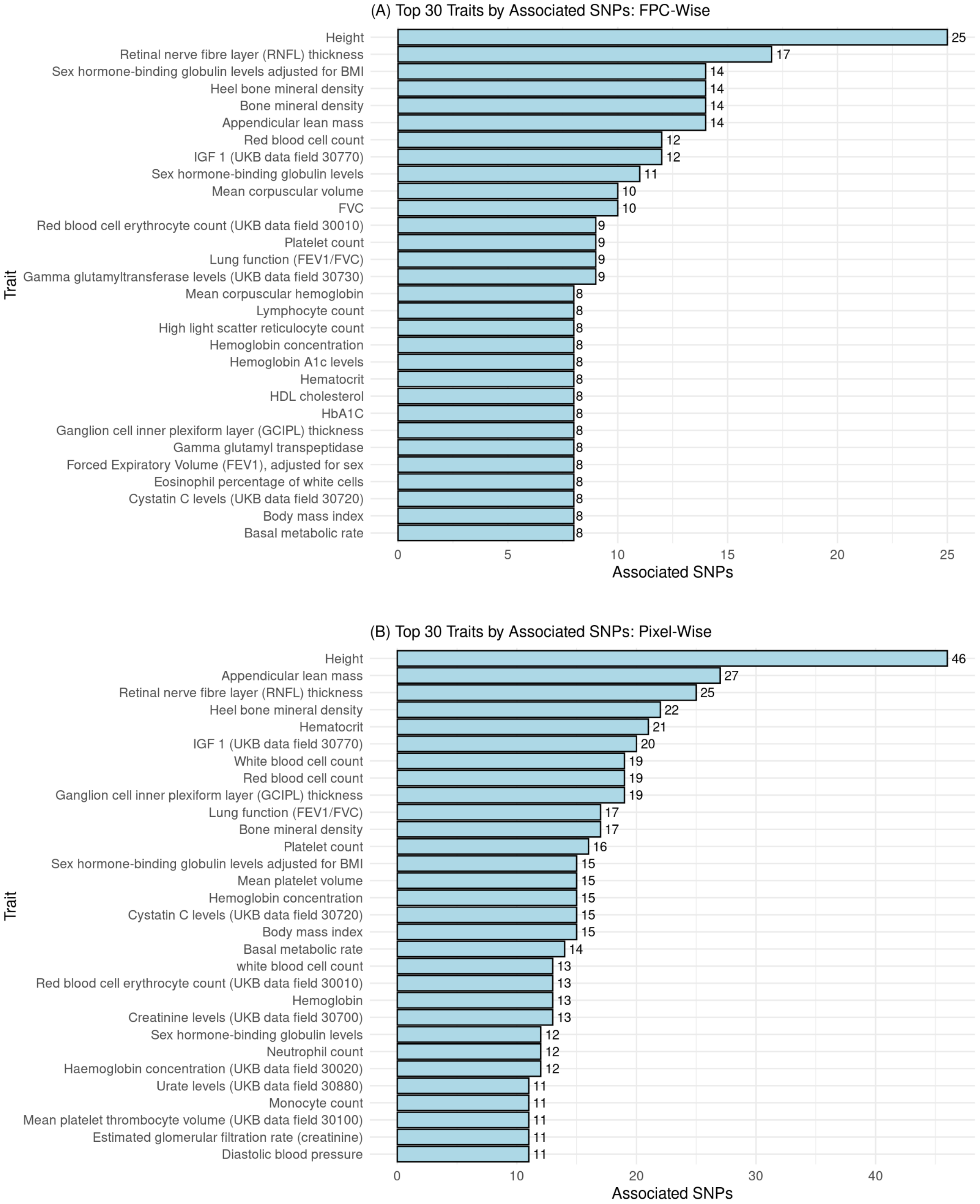
(A) Top 30 Traits by Associated SNPs: FPC-Wise
Trait
Height 25
Retinal nerve fibre layer (RNFL) thickness 17
Sex hormone-binding globulin levels adjusted for BMI 14
Heel bone mineral density 14
Bone mineral density 14
Appendicular lean mass 14
Red blood cell count 12
IGF 1 (UKB data field 30770) 12
Sex hormone-binding globulin levels 11
Mean corpuscular volume 10
FVC 10
Red blood cell erythrocyte count (UKB data field 30010) 9
Platelet count 9
Lung function (FEV1/FVC) 9
Gamma glutamyltransferase levels (UKB data field 30730) 9
Mean corpuscular hemoglobin 8
Lymphocyte count 8
High light scatter reticulocyte count 8
Hemoglobin concentration 8
Hemoglobin A1c levels 8
Hematocrit 8
HDL cholesterol 8
HbA1C 8
Ganglion cell inner plexiform layer (GCIPL) thickness 8
Gamma glutamyl transpeptidase 8
Forced Expiratory Volume (FEV1), adjusted for sex 8
Eosinophil percentage of white cells 8
Cystatin C levels (UKB data field 30720) 8
Body mass index 8
Basal metabolic rate 8
0 5 10 15 20 25
Associated SNPs
(B) Top 30 Traits by Associated SNPs: Pixel-Wise
Trait
Height 46
Appendicular lean mass 27
Retinal nerve fibre layer (RNFL) thickness 25
Heel bone mineral density 22
Hematocrit 21
IGF 1 (UKB data field 30770) 20
White blood cell count 19
Red blood cell count 19
Ganglion cell inner plexiform layer (GCIPL) thickness 19
Lung function (FEV1/FVC) 17
Bone mineral density 17
Platelet count 16
Sex hormone-binding globulin levels adjusted for BMI 15
Mean platelet volume 15
Hemoglobin concentration 15
Cystatin C levels (UKB data field 30720) 15
Body mass index 15
Basal metabolic rate 14
white blood cell count 13
Red blood cell erythrocyte count (UKB data field 30010) 13
Hemoglobin 13
Creatinine levels (UKB data field 30700) 13
Sex hormone-binding globulin levels 12
Neutrophil count 12
Haemoglobin concentration (UKB data field 30020) 12
Urate levels (UKB data field 30880) 11
Monocyte count 11
Mean platelet thrombocyte volume (UKB data field 30100) 11
Estimated glomerular filtration rate (creatinine) 11
Diastolic blood pressure 11
0 10 20 30 40
Associated SNPs

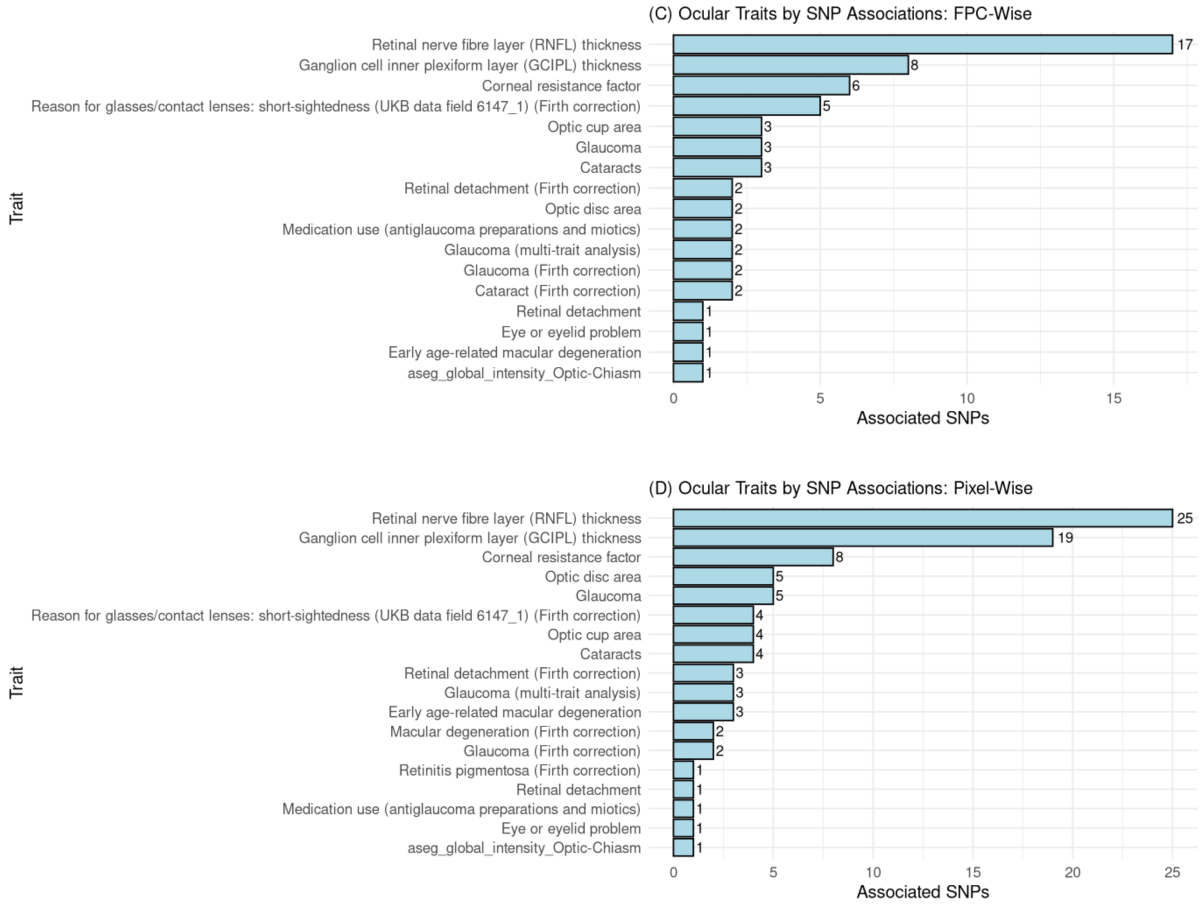

Supplementary Figure 23. Top 30 traits by count of SNP associations from PheWAS for SNPs identified by FPC (A) and pixel-wise (B) analyses. Top SNP-ocular trait association for SNPs identified by FPC (C) and pixel-wise (D) analyses.

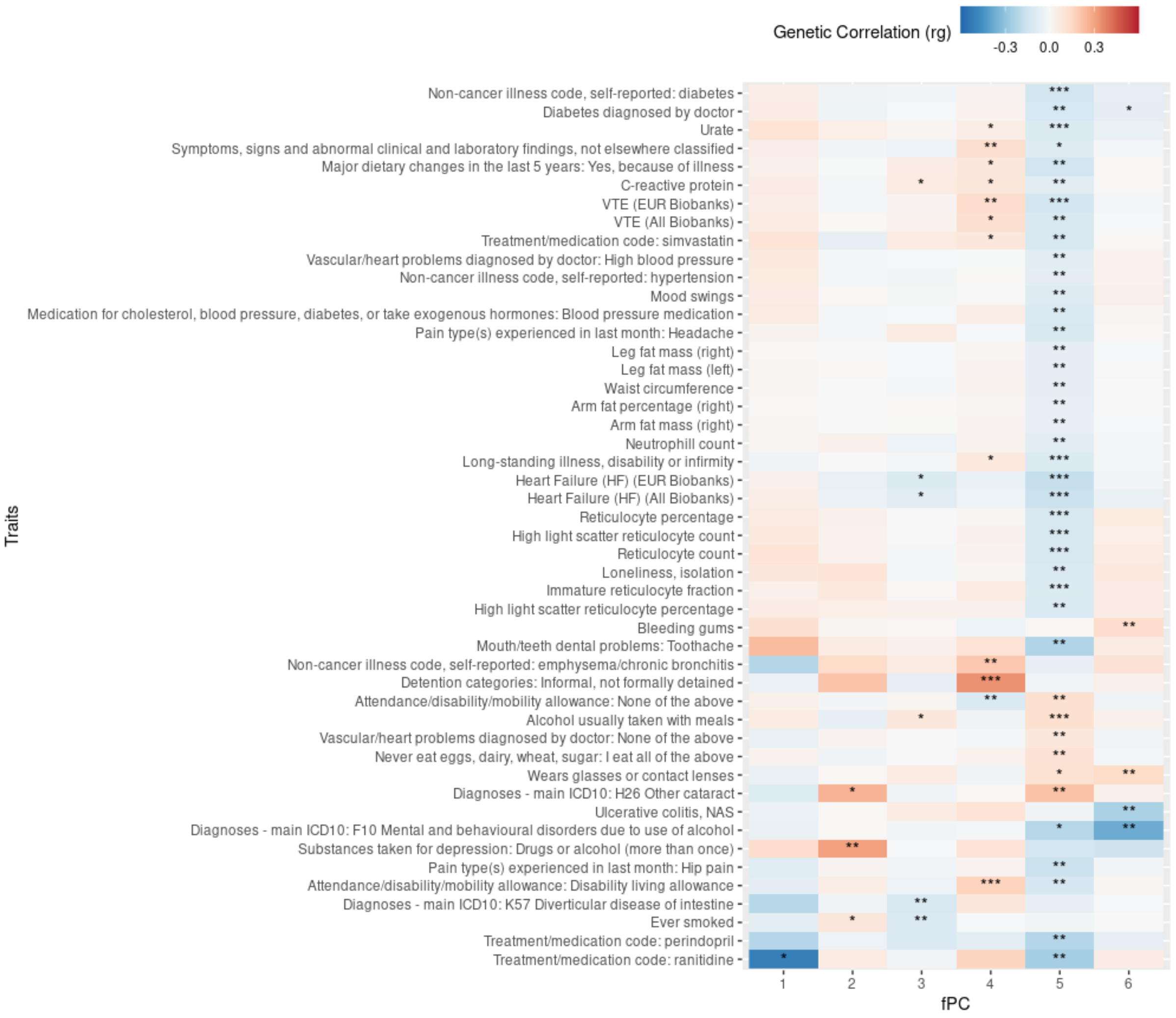

Supplementary Figure 24. Genetic correlations

Traits showing the most statistically significant genetic correlations (rg), estimated using LD-score regression, with RT FPCs 1-6. Significance of genetic correlation: * P < 0.05; ** P < 0.005; *** P < 0.0005 (p-values based on a two-tailed test of a Z-statistic).

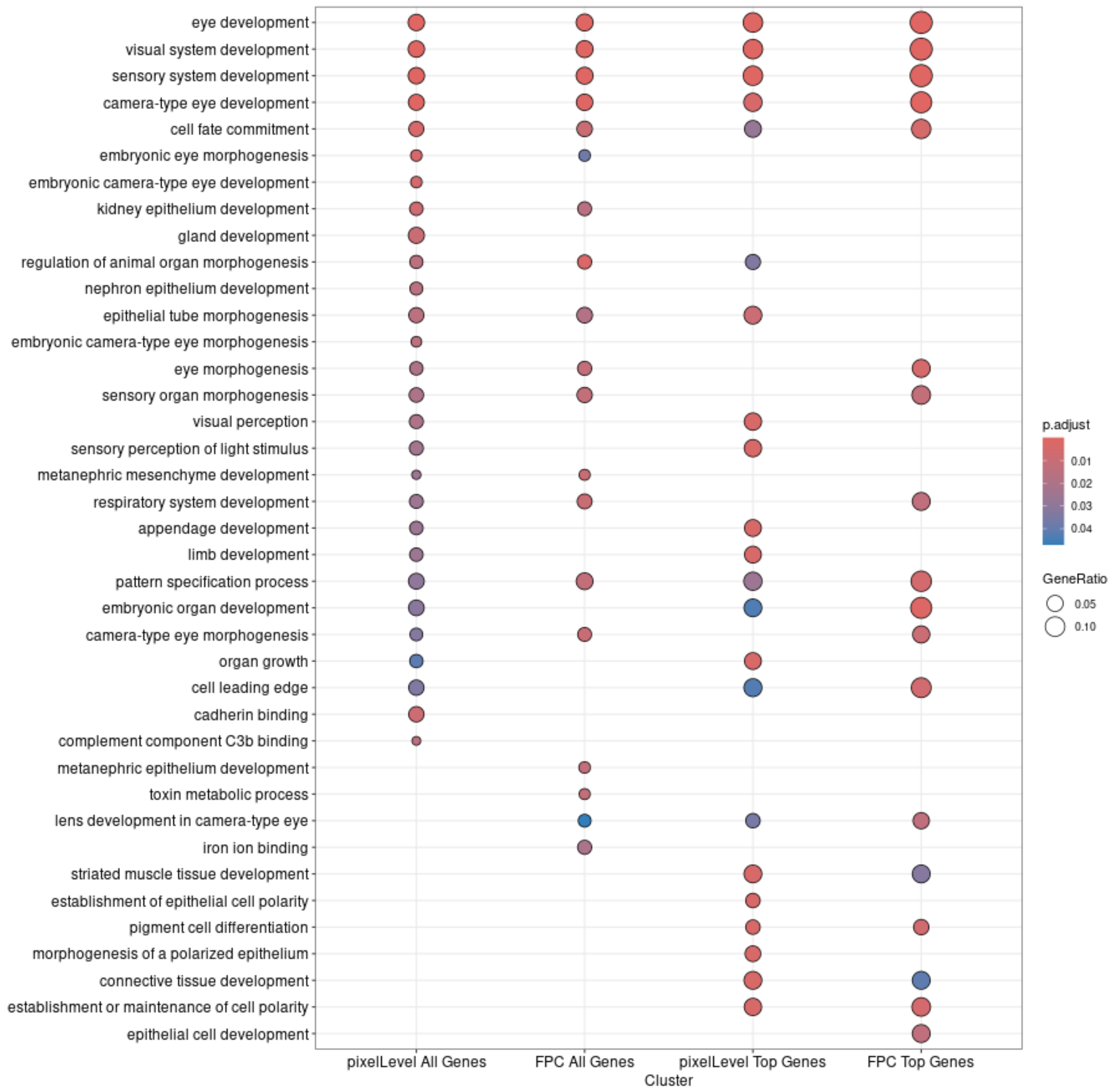

Supplementary Figure 25. Gene Ontology Over-Representation Analysis
Shown are gene Ontology, over-enrichment analysis results for four gene sets: all candidate genes implicated via the pixel-level and fPC locus to gene mapping, and the “Top genes” for each analysis, defined as the gene(s) with the most lines of evidence for each locus (i.e. highest “evidenceScore”, Supplementary Data 6). Shown are Benjamini-Hochberg adjusted p-values for over-representation; p-values calculated using a hypergeometric distribution.

A.

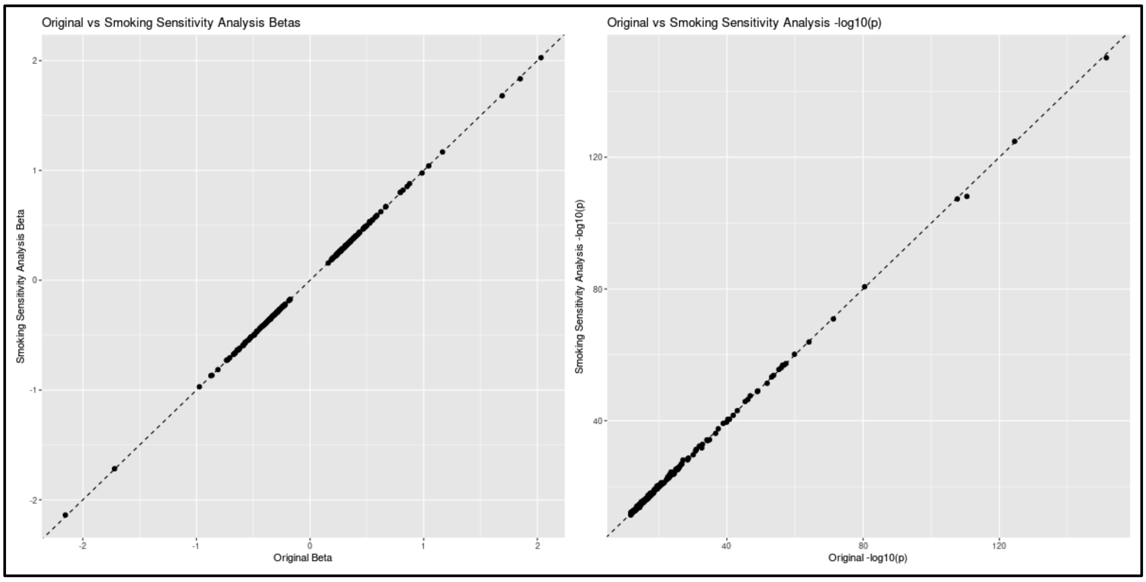

B.

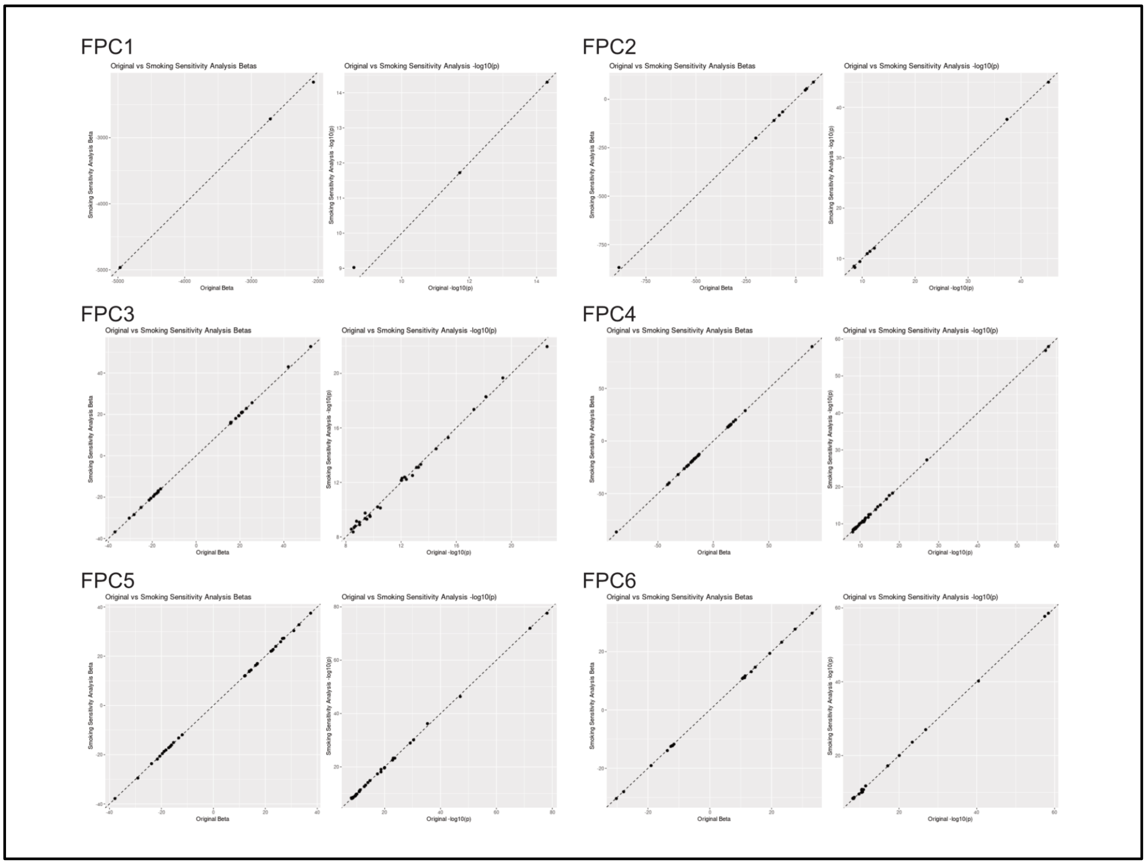

Supplementary Figure 26. Smoking Sensitivity analyses. Comparison of effect estimates and -log10 P-values for SNPs identified as meeting the Bonferroni corrected significance threshold in: A) the pixel-level analyses and B) GWAS of FPCs 1 to 6. P-values based on a two-sided t-test, for the SNP beta in the linear regression.

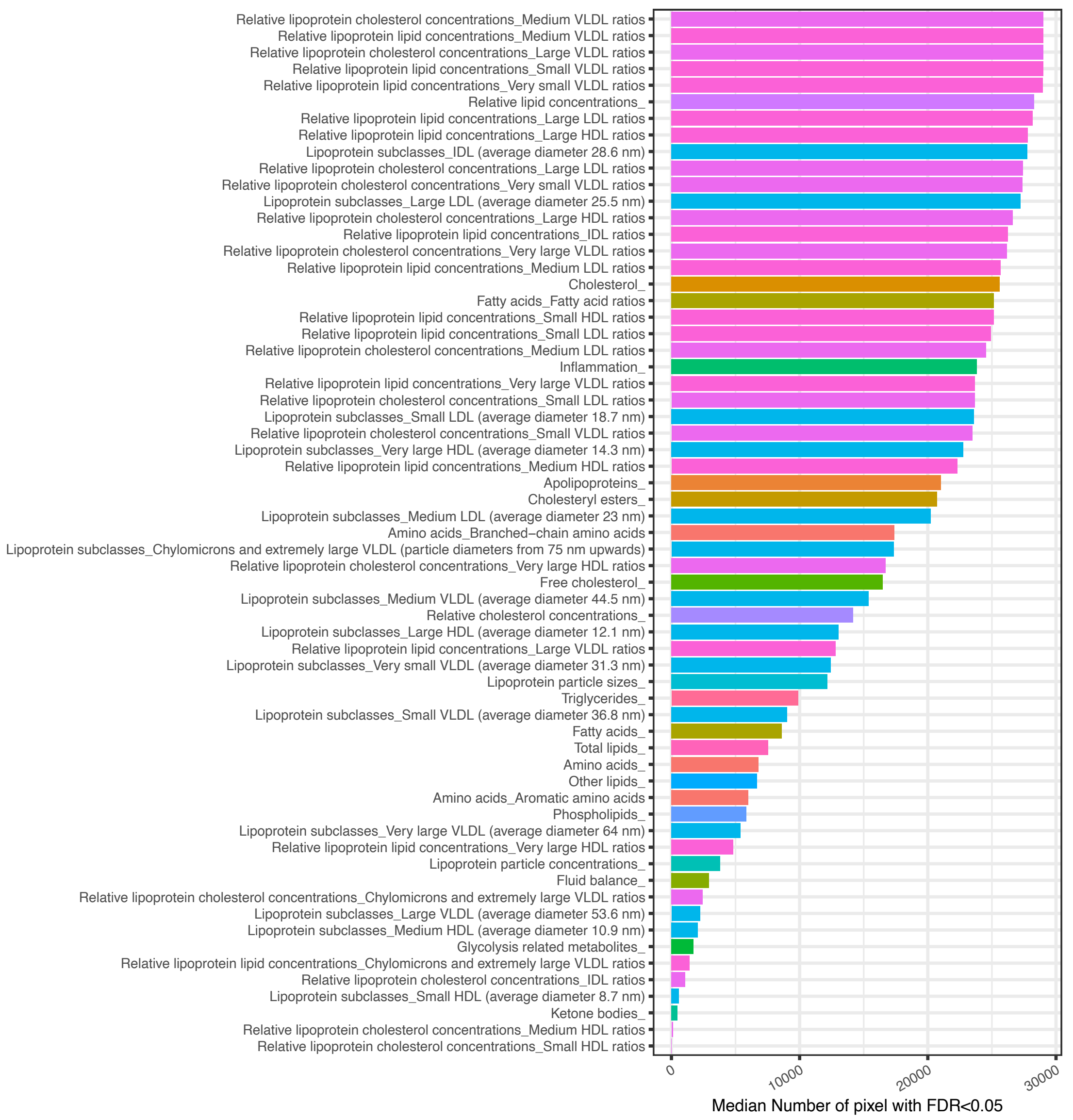

Supplementary Figure 27. Pixel-level metabolite associations
Barplot showing the median number of pixels whose thickness was affected by metabolite in each metabolic group.

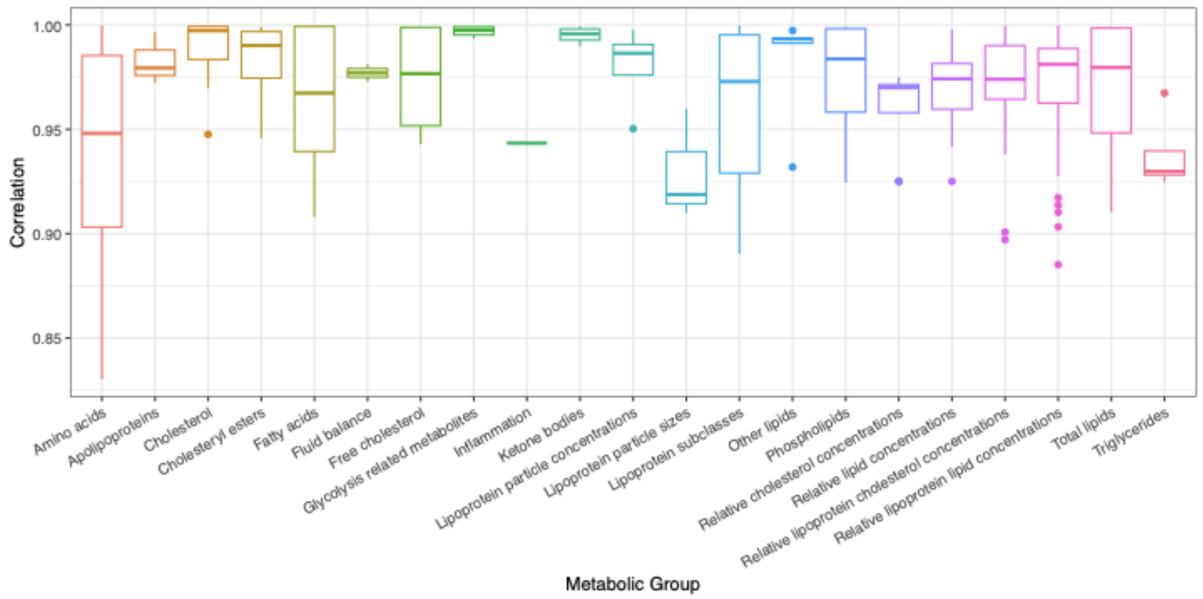

Supplementary Figure 28. BMI sensitivity analyses for metabolite associations.
Correlations of effect estimates across all pixels, for each metabolite (divided into metabolic groups) derived from models correcting and not correcting for BMI.
For each metabolite group, the centre line represents the median value, the box limits correspond to the interquartile range (IQR; 25th to 75th percentiles), whiskers extend to the smallest and largest values within 1.5 times the IQR, with points beyond the whiskers representing outliers.

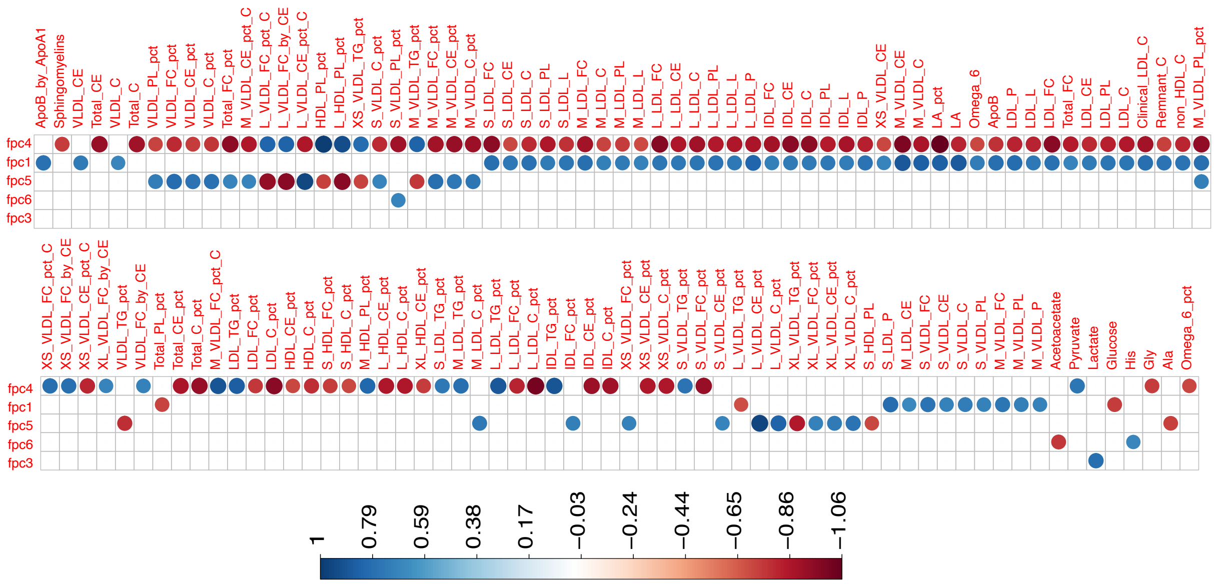

Supplementary Figure 29. Metabolite associations with FPCs
Heatmap showing significant association between metabolic measures and thickness FPCs. Color represents effect estimate direction and magnitude (red=negative, blue=positive). Size represents the magnitude of effect. Full metabolites names available in Supplementary Data 13.

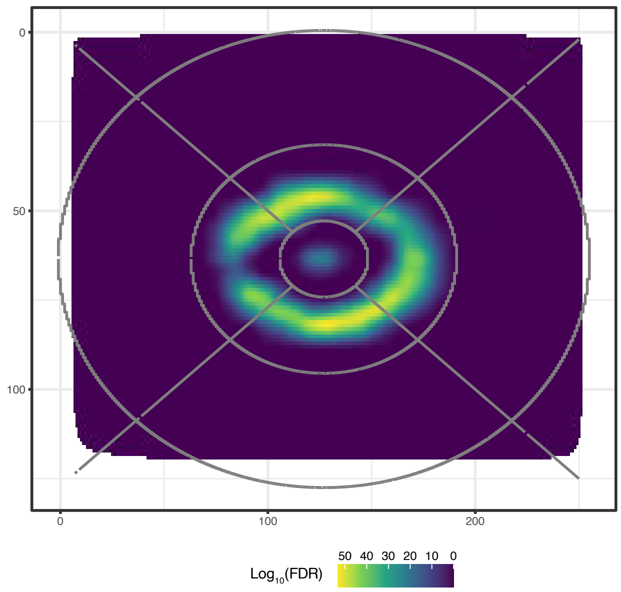

Supplementary Figure 30. Over-representation analysis on age-interaction metabolite results.
2D smoothed results from ORA analysis on age-interaction metabolic results.Shown are Benjamini-Hochberg adjusted p-values for over-representation; p-values calculated using a hypergeometric distribution.

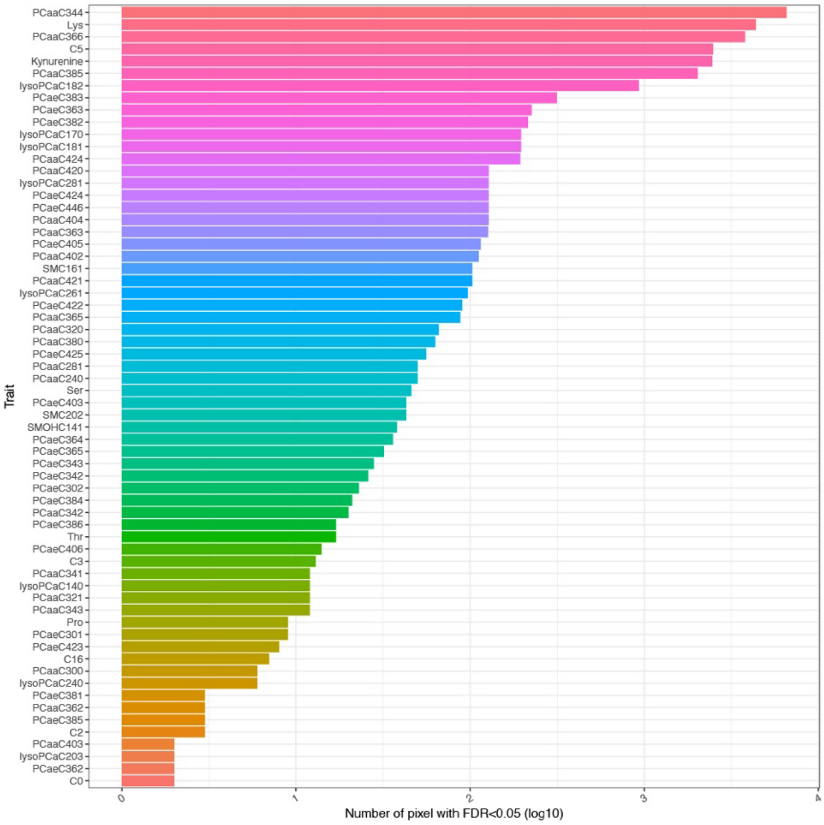

Supplementary Figure 31. Metabolite Genetic Score associations
Barplot showing the count of pixels significantly associated with each metabolic genetic score (full metabolites names available in Supplementary Data 15).

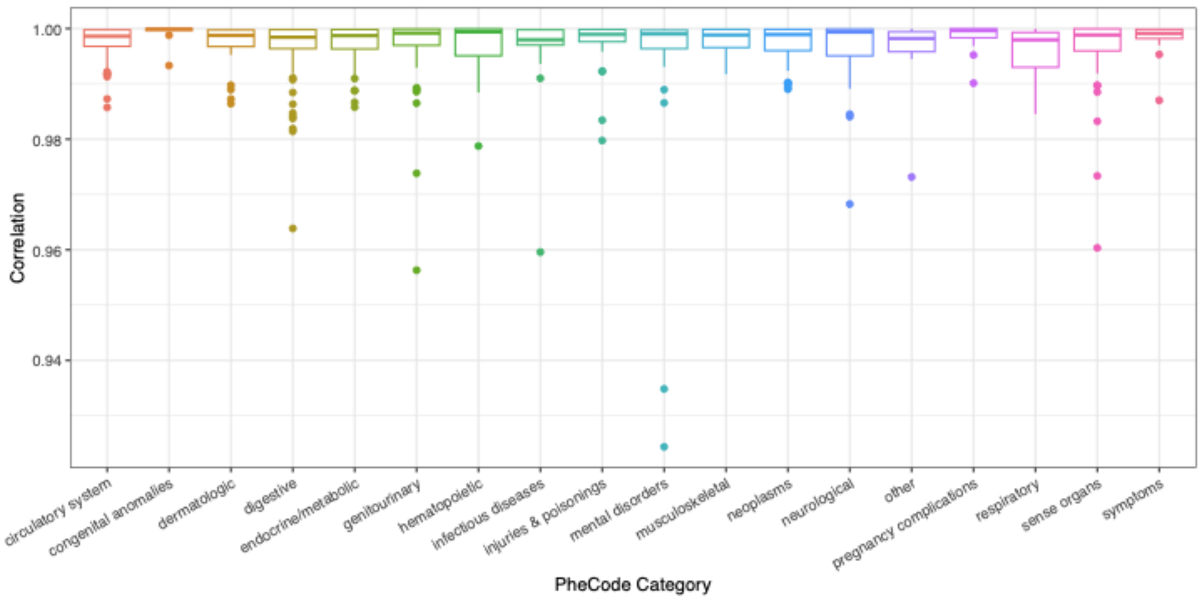

Supplementary Figure 32. Smoking sensitivity analysesfor PheCode associations
Correlations of effect estimates across all pixels, for each metabolite (divided into PheCode groups) derived from models correcting and not correcting for smoking.
For each PheCode category, the centre line represents the median value, the box limits correspond to the interquartile range (IQR; 25th to 75th percentiles), whiskers extend to the smallest and largest values within 1.5 times the IQR, with points beyond the whiskers representing outliers.

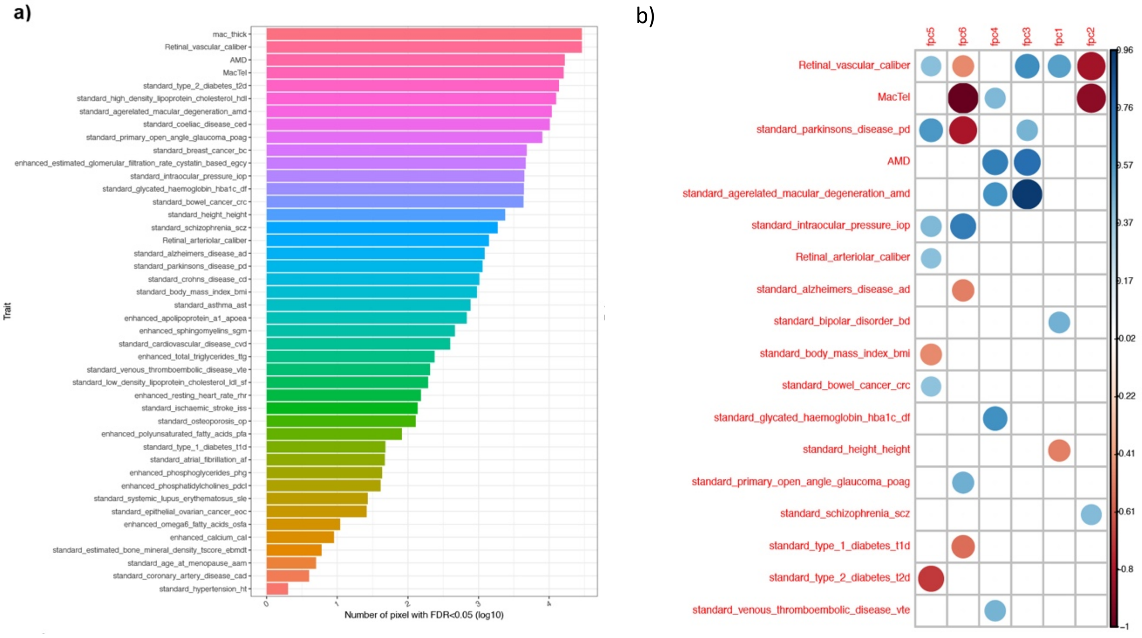

Supplementary Figure 33. Disease and quantitative trait Genetic Score associations
A) Barplot showing the count of pixels significantly associated with each genetic score. B) Heatmap showing significant association between trait genetic scores and thickness FPCs (average retinal thickness PRS removed given extreme effect on all FPCs). Color represents effect estimate direction and magnitude (red=negative, blue=positive). Size represents the magnitude of effect.

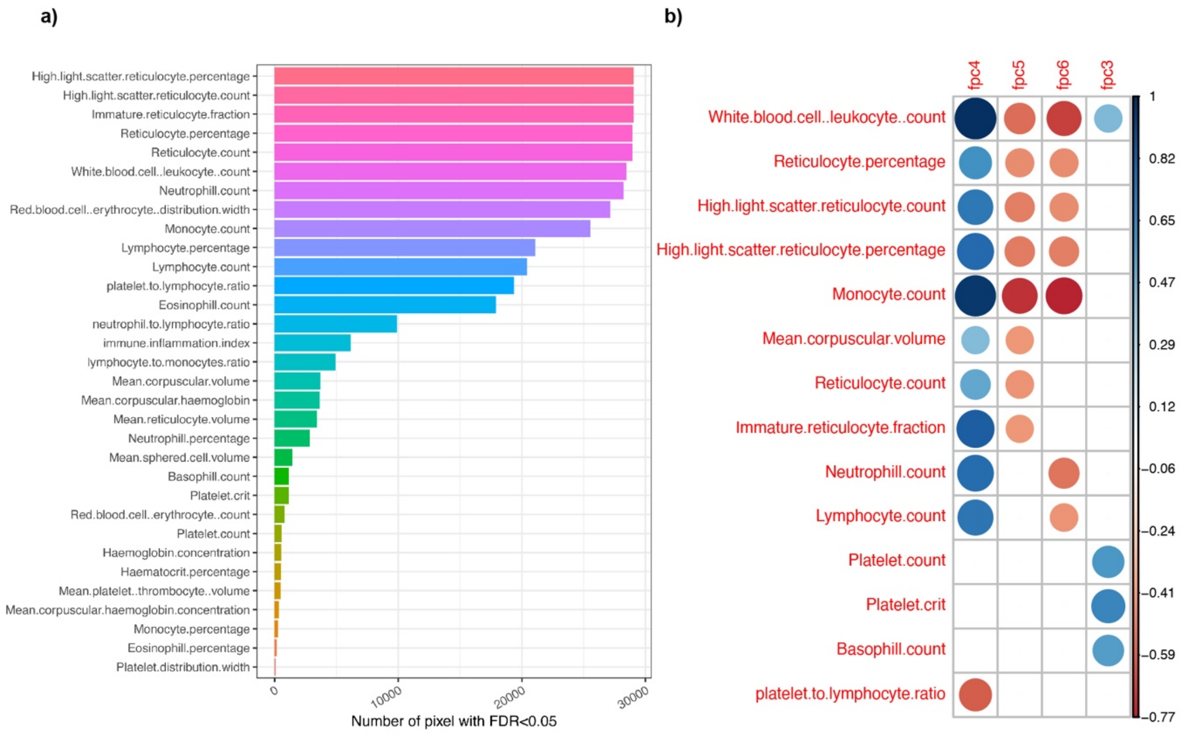

Supplementary Figure 34. Associations with blood cell traits and inflammation markers A) Barplot showing the number of pixels count of pixels significantly associated with each blood or inflammation marker. B) Heatmap showing significant association between significant blood / inflammation marker and thickness FPCs. Color represents effect estimate direction and magnitude (red=negative, blue=positive). Size represents the magnitude of effect.

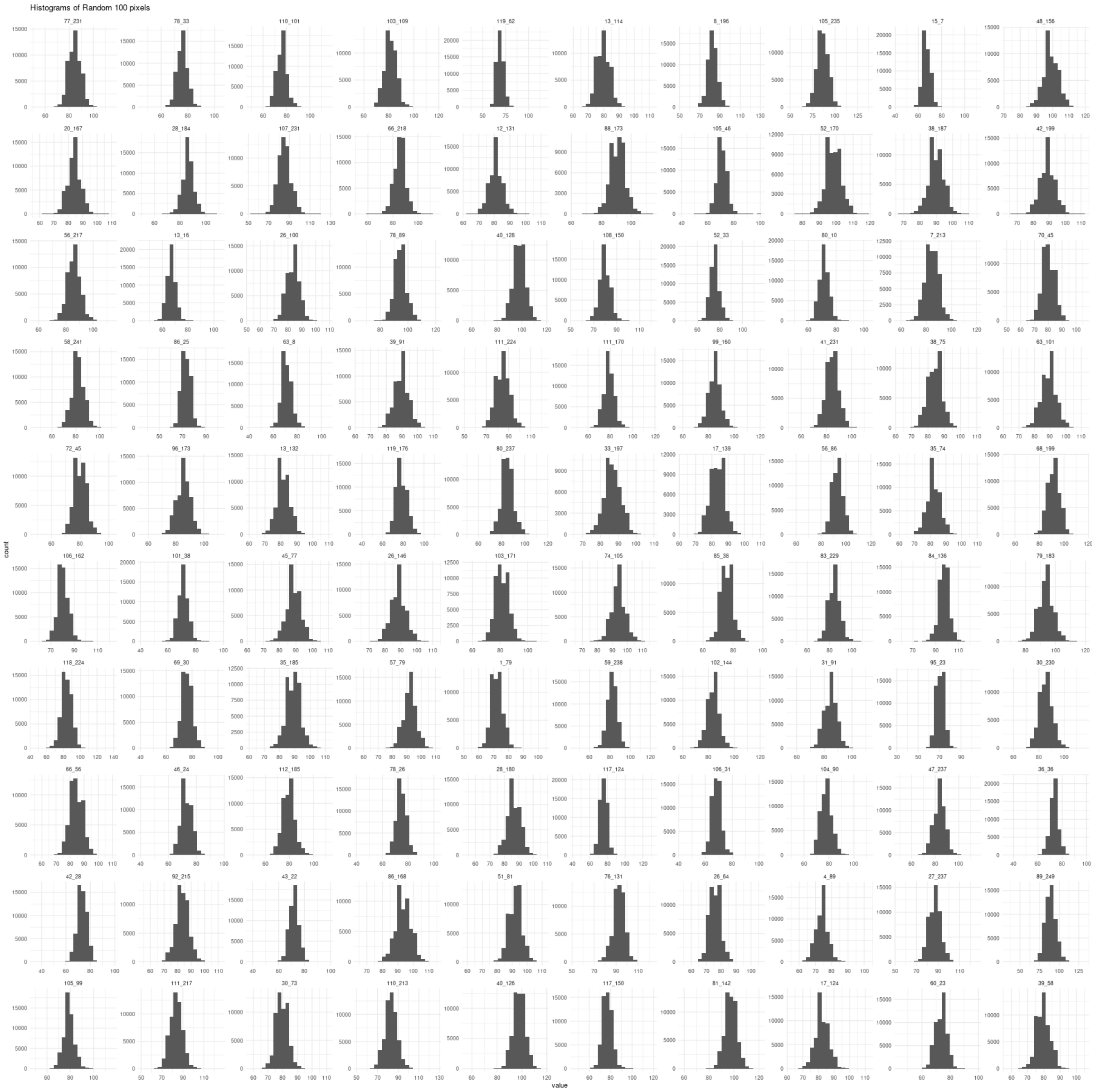

Supplementary Figure 35. Distributions of 100 randomly selected pixels.

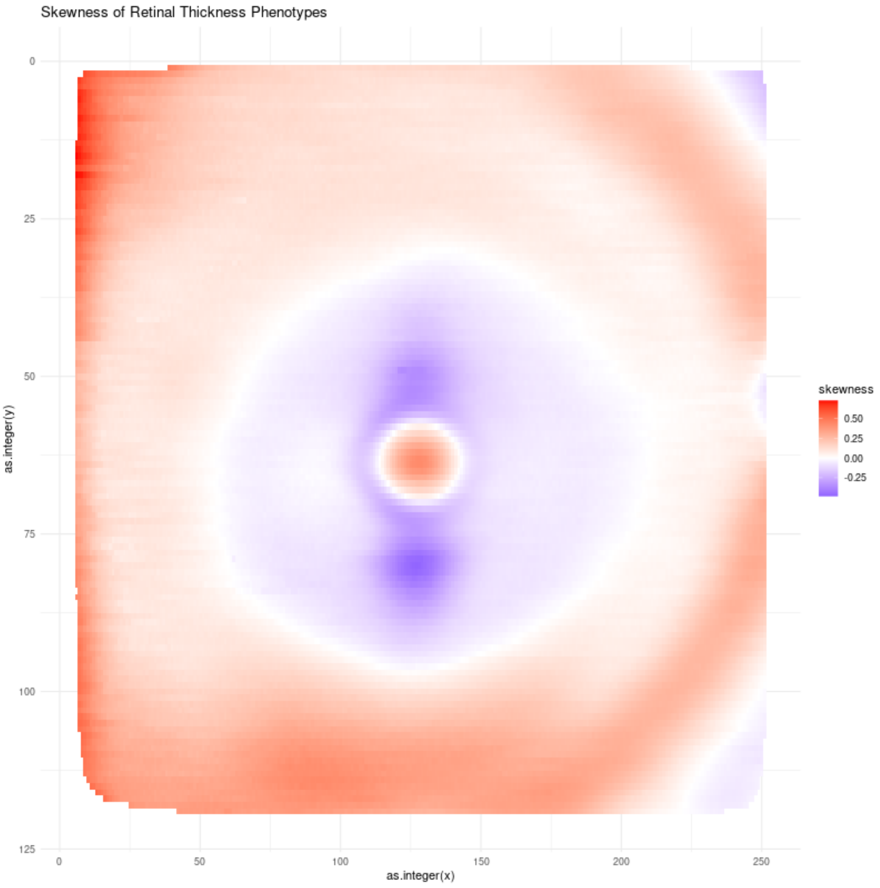

Supplementary Figure 36: Pearson's moment coefficient of skewness for each pixel across the scan

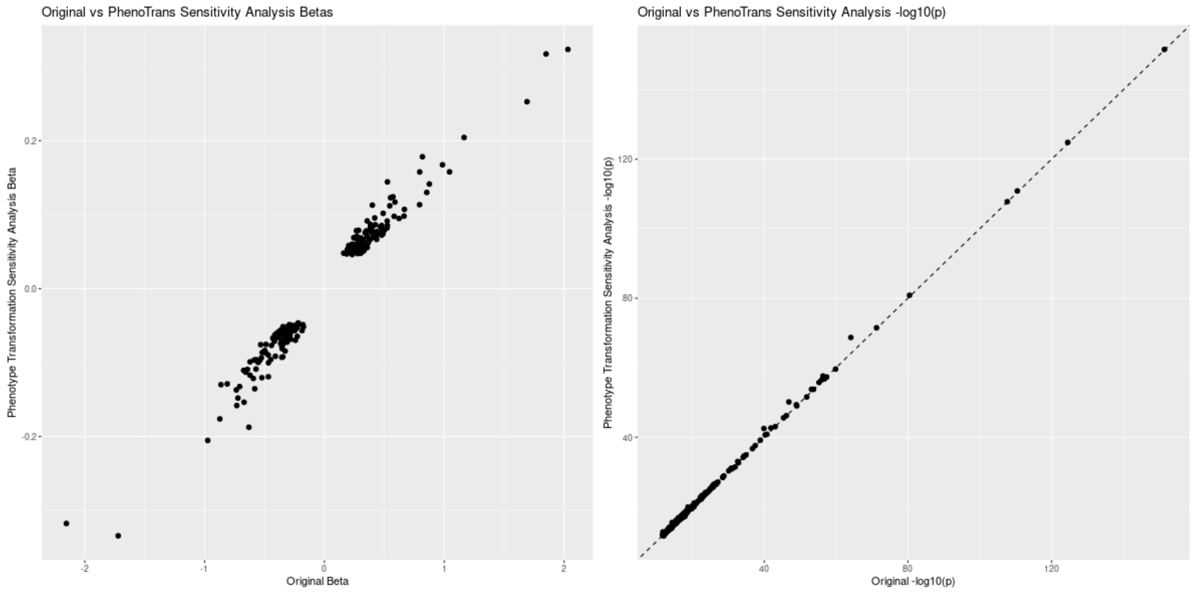

Supplementary Figure 37: Sensitivity analyses for pixel-level RT trait transformations Comparison of effect estimates and -log10 P-values for reported SNPs, based on associations of raw RT phenotypes (x) versus rank inverse normal transformed phenotypes (y). P-values based on a two-sided t-test, for the SNP beta in the linear regression.

Supplementary Tables

Supplementary Table 1. Available OCT data, and participant exclusions

		Individuals	Scans
Scans processed by segmentation pipeline, and passing all ML QC.		***76,998***	***133,460***
Individuals with with eye-related conditions identified based on health care records	ICD10 - 482 relevant codes; ICD9 - 420 relevant codes	19,583	
Individuals taking treatment/medication for retinal diseases	medication for serious eye disease (glaucoma, uveitis, keratitis, uveitis) - 243 relevant codes		
Individuals with self-reported eye conditions	UK Biobank field IDs: 4689: "Age glaucoma diagnosed"; 4700: "Age cataract diagnosed"; 5430: "Age when loss of vision due to injury or trauma diagnosed"; 5901: "Age when diabetes-related eye disease diagnosed"; 5923: "Age macular degeneration diagnosed"; 5945: "Age other serious eye condition diagnosed"; 5408: "Which eye(s) affected by amblyopia (lazy eye)"; 5419: "Which eye(s) affected by injury or trauma resulting in loss of vision"; 5877: "Which eye(s) affected by other eye condition"; 5890: "Which eye(s) affected by diabetes-related eye disease"; 5912: "Which eye(s) affected by macular degeneration"; 5934: "Which eyes affected by other serious eye condition"; 6119: "Which eye(s) affected by glaucoma"; 6205: "Which eye(s) affected by strabismus (squint)"; 6148: "Eye problems/disorders"		
Eyes identified as diseased as part of ML QC			501
Scans deemed to be outliers from manual inspection			251
Individuals without a scan with <10% pixels missing, following pixel-level QC		1716	
Individuals without refractive error measurements carried out at the same time as their OCT scans		494	
Final exclusion of individuals with outlier scan data, following imputation and averaging of eyes (if applicable)		111	
Cleaned Scans data-set		***54,844***	***36,653 (average of both eyes); 18,191 (single eye only)***

Supplementary Table 2. Basic characteristics for the analysed cohort

	EUR	CSA	AFR
N individuals	43,148	1179	1161
Sex (% male)	47.10%	51.10%	40.40%
Spherical Equivalent mean (SD)	-0.24 (2.34)	-0.51 (2.24)	-0.36 (1.98)
Age Mean (SD)	57.0 (8.08)	51.3 (8.15)	51.1 (7.27)
Standing Height cm Mean (SD)	169.4 (9.12)	164.2 (9.15)	167.7 (8.52)
Scan-wise mean thickness Mean (SD)	83.15 (3.64)	81.43 (3.54)	81.19 (3.66)
Scan-wise minimum thickness Mean (SD)	60.844 (3.94)	58.34 (3.71)	57.30 (3.87)
Scan-wise maximum thickness Mean (SD)	103.42 (5.06)	103.72 (6.26)	101.87 (6.05)

Supplementary Table 3. Associations between RT fPCs and basic characteristics

variable	beta_fpc1	pVal_fpc1	beta_fpc2	pVal_fpc2	beta_fpc3	pVal_fpc3	beta_fpc4	pVal_fpc4	beta_fpc5	pVal_fpc5	beta_fpc6	pVal_fpc6
age	119.22	1.09E-12	0.45	0.959	-13.22	1.20E-05	-19.42	6.93E-14	1.78	0.445	11.03	4.74E-08
age Squared	-1.31	3.03E-18	0.11	0.161	0.05	0.063	0.2	2.17E-17	0	0.822	-0.11	3.46E-10
Spherical Equivalent	177.16	<2.23E-308	-139.3	<2.23E-308	8.78	1.86E-29	-17.67	7.75E-153	-36.76	<2.23E-308	-23.51	<2.23E-308
Sex - Male (vs Female)	-138.45	1.37E-06	-158.21	1.32E-25	-154.38	3.61E-194	-167.39	1.04E-306	35.1	1.43E-18	-7.85	0.023
Standing Height	6.7	2.16E-05	2.09	0.012	0.45	0.115	-1.28	1.66E-07	3.49	6.24E-57	-0.1	0.615
Ancestry - AFR (vs EUR)	-78.14	0.215	-306.22	3.17E-20	-129.99	2.78E-30	70.85	3.78E-13	-325.19	8.70E-297	-92.66	3.89E-34
Ancestry - CSA (vs EUR)	-97.39	0.125	-159.08	1.97E-06	15.18	0.184	78.8	1.02E-15	-178.49	1.39E-90	29.92	9.18E-05

Betas and p-values (pVal) from the linear regression model. P-values based on a two-sided t-test, for the corresponding beta.

Supplementary Table 4. Sample sizes for -omic association analyses

	EUR	AFR	CSA
**Genetics**	43,148	1,179	1,161
**Metabolomics**	10,668	not conducted	not conducted
**Immunomics & Blood traits (Inflammation markers)**	39,611	not conducted	not conducted
**Infection antigens**	764	not conducted	not conducted
**ICD10 codes**	36,196	not conducted	not conducted
**PRS**	43,147	not conducted	not conducted

Supplementary References

1. Currant, H. *et al.* Genetic variation affects morphological retinal phenotypes extracted from UK Biobank optical coherence tomography images. *PLoS Genet.* **17**, e1009497 (2021).
2. Currant, H. *et al.* Sub-cellular level resolution of common genetic variation in the photoreceptor layer identifies continuum between rare disease and common variation. *PLoS Genet.* **19**, e1010587 (2023).
3. Gao, X. R., Huang, H. & Kim, H. Genome-wide association analyses identify 139 loci associated with macular thickness in the UK Biobank cohort. *Hum. Mol. Genet.* **28**, 1162–1172 (2019).
4. Zekavat, S. M. *et al.* Phenome- and genome-wide analyses of retinal optical coherence tomography images identify links between ocular and systemic health. *Sci. Transl. Med.* **16**, eadg4517 (2024).
