# Supplementary material for: Multi-omic spatial effects on high-resolution AI-derived retinal thickness
Source: Nat Commun. 2025 Feb 4;16:1317. doi: 10.1038/s41467-024-55635-7 (PMC11794613; doi:10.1038/s41467-024-55635-7)
Supplement: Supplementary file 2 — Description of Additional Supplementary Files [file 41467_2024_55635_MOESM2_ESM.pdf]

Supplementary Data Index

Sheet Name: Column Descriptors A
Description: Column descriptors for Supplementary Data 1-12

Sheet Name: Column Descriptors B
Description: Column descriptors for Supplementary Data 13-20

Sheet Name: SupplementaryData1
Description: Independent loci identified through the pixel-level RT GWAS

Sheet Name: SupplementaryData2
Description: Independent loci identified through the FPC RT GWAS

Sheet Name: SupplementaryData3
Description: Mapping of sentinel SNPs and proxies (r2 >0.5) to genes, based on genomic postition.

Sheet Name: SupplementaryData4
Description: Mapping of sentinel SNPs and proxies (r2 >0.5) to genes, based on co-localisation of eQTLs from retina, blood and brain tissue.

Sheet Name: SupplementaryData5
Description: Mapping of sentinel SNPs and proxies (r2 >0.5) to genes, based on chromatin interaction data from adut and fetal cortex, and retina

Sheet Name: SupplementaryData6
Description: Summary of all genes mapped from GWAS loci, based on positional, eQTL , or chromatin Interaction mapping.

Sheet Name: SupplementaryData7
Description: Look up of loci identified in the pixel-level analyses in previous GWAS of retinal thickness traits

Sheet Name: SupplementaryData8
Description: Look up of loci identified in the FPC analyses in previous GWAS of retinal thickness traits

Sheet Name: SupplementaryData9
Description: Comparison of effects in European, Central and South Asian and African ancestries, for all sentinel SNPs identified through the pixel-level, or FPC GWAS.

Sheet Name: SupplementaryData10

Description: PheWAS results for all identified SNPs.

Sheet Name: SupplementaryData11
Description: Results of genetic correlation analysis using LD-score regression
Sheet Name: SupplementaryData12
Description: Gene Ontology Over-representation analyses, for genes identified through the fPC and pixel-level GWAS

Sheet Name: SupplementaryData13
Description: Metabolite information and their average association statistics with retinal thickness

Sheet Name: SupplementaryData14
Description: Metabolite information and their average interaction effect with age on retinal thickness.

Sheet Name: SupplementaryData15
Description: Metabolic genetic score information and their average association statistics with retinal thickness

Sheet Name: SupplementaryData16
Description: PheCode information and their average association statistics with retinal thickness

Sheet Name: SupplementaryData17
Description: List of trait genetic scores, their information and their average association statistics with retinal thickness.

Sheet Name: SupplementaryData18
Description: Analysis of correlation across pixels between disease Phecodes and respective genetic scores

Sheet Name: SupplementaryData19
Description: Blood and inflammation marker information and their average association statistics with retinal thickness

Sheet Name: SupplementaryData20
Description: Antigen markers of infection information and their average association statistics with retinal thickness
