# Supplementary material for: Multi-omic spatial effects on high-resolution AI-derived retinal thickness
Source: Nat Commun. 2025 Feb 4;16:1317. doi: 10.1038/s41467-024-55635-7 (PMC11794613; doi:10.1038/s41467-024-55635-7)
Supplement: Supplementary file 4 — Reporting Summary [file 41467_2024_55635_MOESM4_ESM.pdf]

nature portfolio

Corresponding author(s): Melanie Bahlo

Last updated by author(s): 2024-11-22

Reporting Summary

Nature Portfolio wishes to improve the reproducibility of the work that we publish. This form provides structure for consistency and transparency in reporting. For further information on Nature Portfolio policies, see our Editorial Policies and the Editorial Policy Checklist.

Statistics

For all statistical analyses, confirm that the following items are present in the figure legend, table legend, main text, or Methods section.

n/a	Confirmed	
☐	☒	The exact sample size (*n*) for each experimental group/condition, given as a discrete number and unit of measurement
☐	☒	A statement on whether measurements were taken from distinct samples or whether the same sample was measured repeatedly
☐	☒	The statistical test(s) used AND whether they are one- or two-sided<br>*Only common tests should be described solely by name; describe more complex techniques in the Methods section.*
☐	☒	A description of all covariates tested
☐	☒	A description of any assumptions or corrections, such as tests of normality and adjustment for multiple comparisons
☐	☒	A full description of the statistical parameters including central tendency (e.g. means) or other basic estimates (e.g. regression coefficient) AND variation (e.g. standard deviation) or associated estimates of uncertainty (e.g. confidence intervals)
☐	☒	For null hypothesis testing, the test statistic (e.g. *F*, *t*, *r*) with confidence intervals, effect sizes, degrees of freedom and *P* value noted<br>*Give P values as exact values whenever suitable.*
☒	☐	For Bayesian analysis, information on the choice of priors and Markov chain Monte Carlo settings
☒	☐	For hierarchical and complex designs, identification of the appropriate level for tests and full reporting of outcomes
☐	☒	Estimates of effect sizes (e.g. Cohen's *d*, Pearson's *r*), indicating how they were calculated

Our web collection on statistics for biologists contains articles on many of the points above.

Software and code

Policy information about availability of computer code

Data collection	No software were used for data collection.
Data analysis	Deep learning algorithm was implemented in Python3.7 and PyTorch0.4.<br>Deep learning QC was done with bespoke code in Python3.6+, opencv4.5+, scikit-learn0.24+, numpy1.19+<br><br>Software used for genetic analyses: QCTool (v2); Plink2 (version 20221024); Plink (v1.90b6.18); R (v.4.1.2); VEP (v.109); FUMA (v1.5.3); ANNOVAR (2017-07-17); Complex Traits Genetics Virtual Lab (https://vl.genoma.io/); LD-score regression (v.1);<br><br>R packages utilised in data-cleaning, genetics and multi-omic analyses: mgcv (v.1.8-40); MFPCA (v. 1.3-10); ClusterProfiler (v. 4.2.2); bigSNPR (version 1.12.2); ukbnmr (version 1.5); MICE (version 3.15.0); ukbtool (version 0.11.3); icd (version 4.0.9); limma (version 3.50.3); magrittr; data.table; dplyr; ggplot2; here; DescTools; funData; purrr; patchwork; RColorBrewer; GGally; rlist; doparallel; foreach; abind; stringr; UpSetR; ggside; enrichplot; dendextend; scales; pheatmap; tidyverse; org.Hs.eg.db; lubridate; coloc (version 5.2.3); SuSiE (version 0.12.4); ieugwasr (version 1.0.0); ggmanh (version 3.20)<br><br>Scripts for the genetic analyses are available at https://doi.org/10.5281/zenodo.14202924

For manuscripts utilizing custom algorithms or software that are central to the research but not yet described in published literature, software must be made available to editors and reviewers. We strongly encourage code deposition in a community repository (e.g. GitHub). See the Nature Portfolio guidelines for submitting code & software for further information.

Data

Policy information about availability of data

All manuscripts must include a data availability statement. This statement should provide the following information, where applicable:

- Accession codes, unique identifiers, or web links for publicly available datasets
- A description of any restrictions on data availability
- For clinical datasets or third party data, please ensure that the statement adheres to our policy

GWAS summary statistics for the six 2D FPCs, and pixel-level results for all 'omics and disease code analyses, are available to download from https://doi.org/10.17605/OSF.IO/KZUGV.

Summary statistics for the six FPC GWAS have also been uploaded to the GWAS catalogue, under accession numbers GCST90455721- GCST90455726.

All association results(including genetics) at the pixel level are accessible through a bespoke web browser (https://retinomics.org/).

Access to individual level UK Biobank (https://www.ukbiobank.ac.uk/), data is available to bona fide researchers upon application (in accordance with the terms of ethical approval and participant consent).

Additionally utilised publicly available datasets: International Mouse Phenotyping consortium (https://www.mousephenotype.org); OMIM database (https://www.omim.org); EyeGEx; GTEx v7 (https://gtexportal.org/home/downloads/adult-gtex/qtl); retina Hi-C dataset (https://www.ncbi.nlm.nih.gov/geo/query/acc.cgi?acc=GSE202471); openGWAS platform (https://gwas.mrcieu.ac.uk/).

Research involving human participants, their data, or biological material

Policy information about studies with human participants or human data. See also policy information about sex, gender (identity/presentation), and sexual orientation and race, ethnicity and racism.

Reporting on sex and gender	Sex was determined using genetic data. We obtained sex information from UK Biobank field Data-Field 22001. Sex was included as a covariate in all analyses.<br>For the primary analyses in this paper, 22,804 (52.9%) individuals were female, 20,344 (47.1%) were male.
Reporting on race, ethnicity, or other socially relevant groupings	Our analyses utilised grouping of individuals by ancestry, based on genetic similarity to individuals from the 1000 Genomes Project and Human Genome Diversity Panel. These broad continental ancestry assignments were generated through the Pan-UK Biobank project (https://pan.ukbb.broadinstitute.org/), and were obtained from UK Biobank returned dataset 2442.
Population characteristics	Amongst the individuals included in the primary analyses in this paper, 47.1% were male, and participants had a mean age of 57.0 (sd=8.1). All individuals were selected as being free of retinal disease or retina related disorders. Genetic information for participants was derived via genotyping with Affymetrix UK BiLEVE or UK Biobank arrays, and imputation to the combined Haplotype Reference Consortium and UK10K panel. All individuals had complete information for the covariates included in the reported analyses: sex; age; age-squared; standing height; OCT imaging device ID; measured eye (left, right, or mean); spherical equivalent (for the corresponding eye(s)); and the first ten ancestry principal components.
Recruitment	The UK Biobank is a prospective cohort study of over 500,000 individuals from across the United Kingdom, aged between 40 and 69 at recruitment. A range of phenotypic information and biological samples have been collected for all participants, in addition to linkage to electronic health records. At baseline, self-reported socio-demographic, lifestyle and health-related factors were collected, along with physical measures, and blood, urine and saliva samples. Follow-up assessment visits, for a subset of participants, included a range of eye measures, including OCT imaging.
Ethics oversight	The UK Biobank genetic and phenotypic data were analysed under UK Biobank Application applications 28541. UK Biobank has ethical approval from the UK National Health Service (NHS) National Research Ethics Service (11/NW/0382). Research was approved by the Walter and Eliza Hall Institute of Medical Research Human Ethics Committee (HREC 17/09LR).

Note that full information on the approval of the study protocol must also be provided in the manuscript.

Field-specific reporting

Please select the one below that is the best fit for your research. If you are not sure, read the appropriate sections before making your selection.

☒ Life sciences ☐ Behavioural & social sciences ☐ Ecological, evolutionary & environmental sciences

For a reference copy of the document with all sections, see nature.com/documents/nr-reporting-summary-flat.pdf

Life sciences study design

All studies must disclose on these points even when the disclosure is negative.

Sample size	The sample size was not pre-determined; instead the analyses utilised the largest available dataset with both OCT imaging data and genome-wide genetic data.
Data exclusions	Analyses were restricted to individuals with both genetic and OCT imaging data available, passing all quality control for these data, and with complete data for utilised covariates (see above).<br>Individuals with evidence of retinal disease or retina related disorders were excluded. Primary analyses included individuals with European ancestries, with secondary analyses in individuals with African and South Asian ancestries. Individuals who were determined to be of any other ancestry, or mixed ancestries were excluded, due to insufficient sample size to allow meaningful analyses.
Replication	No formal replication in independent samples were undertaken, due to unavailability of suitable datasets. Primary genetic analyses were undertaken in individuals with European ancestries; cross-ancestry replication within UK Biobank was undertaken with African and South Asian individuals.

Randomization	This is an observational study, and no randomization was undertaken.
Blinding	No blinding was undertaken for this study. No allocation to groups was made.

Reporting for specific materials, systems and methods

We require information from authors about some types of materials, experimental systems and methods used in many studies. Here, indicate whether each material, system or method listed is relevant to your study. If you are not sure if a list item applies to your research, read the appropriate section before selecting a response.

Materials & experimental systems

n/a	Involved in the study	
☒	☐	Antibodies
☒	☐	Eukaryotic cell lines
☒	☐	Palaeontology and archaeology
☒	☐	Animals and other organisms
☒	☐	Clinical data
☒	☐	Dual use research of concern
☒	☐	Plants

Methods

n/a	Involved in the study	
☒	☐	ChIP-seq
☒	☐	Flow cytometry
☒	☐	MRI-based neuroimaging

Plants

Seed stocks	*Report on the source of all seed stocks or other plant material used. If applicable, state the seed stock centre and catalogue number. If plant specimens were collected from the field, describe the collection location, date and sampling procedures.*
Novel plant genotypes	*Describe the methods by which all novel plant genotypes were produced. This includes those generated by transgenic approaches, gene editing, chemical/radiation-based mutagenesis and hybridization. For transgenic lines, describe the transformation method, the number of independent lines analyzed and the generation upon which experiments were performed. For gene-edited lines, describe the editor used, the endogenous sequence targeted for editing, the targeting guide RNA sequence (if applicable) and how the editor was applied.*
Authentication	*Describe any authentication procedures for each seed stock used or novel genotype generated. Describe any experiments used to assess the effect of a mutation and, where applicable, how potential secondary effects (e.g. second site T-DNA insertions, mosiacism, off-target gene editing) were examined.*
